# The Long-Term Effects of Early Life Stress on the Modulation of miR-19 Levels

**DOI:** 10.3389/fpsyt.2020.00389

**Published:** 2020-05-15

**Authors:** Monica Mazzelli, Carlo Maj, Nicole Mariani, Cristina Mora, Veronica Begni, Carmine M. Pariante, Marco A. Riva, Annamaria Cattaneo, Nadia Cattane

**Affiliations:** ^1^Biological Psychiatry Unit, IRCCS Istituto Centro San Giovanni di Dio Fatebenefratelli, Brescia, Italy; ^2^Institute for Genomic Statistics and Bioinformatics, University Hospital, Bonn, Germany; ^3^Stress, Psychiatry and Immunology Laboratory, Department of Psychological Medicine, Institute of Psychiatry, Psychology and Neuroscience, King’s College London, London, United Kingdom; ^4^Department of Pharmacological and Biomolecular Sciences, University of Milan, Milan, Italy

**Keywords:** early life stress, miR-19, brain trajectories, neurodevelopment, inflammation, depression, schizophrenia

## Abstract

MicroRNAs (miRNAs), one of the major small non-coding RNA classes, have been proposed as regulatory molecules in neurodevelopment and stress response. Although alterations in miRNAs profiles have been implicated in several psychiatric and neurodevelopmental disorders, the contribution of individual miRNAs in brain development and function is still unknown. Recent studies have identified miR-19 as a key regulator of brain trajectories, since it drives the differentiation of neural stem cells into mature neurons. However, no findings are available on how vulnerability factors for these disorders, such as early life stress (ELS), can modulate the expression of miR-19 and its target genes. To reach our aim, we investigated miR-19 modulation in human hippocampal progenitor stem cells (HPCs) treated with cortisol during 3 days of proliferation and harvested immediately after the end of the treatment or after 20 days of differentiation into mature neurons. We also analyzed the long-term expression changes of miR-19 and of its validated target genes, involved in neurodevelopment and inflammation, in the hippocampus of adult rats exposed or not to prenatal stress (PNS). Interestingly, we observed a significant downregulation of miR-19 levels both in proliferating (FC = −1.59, p-value = 0.022 for miR-19a; FC = −1.79, p-value = 0.016 for miR-19b) as well as differentiated HPCs (FC = −1.28, p-value = 0.065 for miR-19a; FC = −1.75, p-value = 0.047 for miR-19b) treated with cortisol. Similarly, we found a long-term decrease of miR-19 levels in the hippocampus of adult PNS rats (FC = −1.35, p-value = 0.025 for miR-19a; FC = −1.43, p-value = 0.032 for miR-19b). Among all the validated target genes, we observed a significant increase of NRCAM (FC = 1.20, p-value = 0.027), IL4R (FC = 1.26, p-value = 0.046), and RAPGEF2 (FC = 1.23, p-value = 0.020).We suggest that ELS can cause a long-term downregulation of miR-19 levels, which may be responsible of alterations in neurodevelopmental pathways and in immune/inflammatory processes, leading to an enhanced risk for mental disorders later in life. Intervention strategies targeting miR-19 may prevent alterations in these pathways, reducing the ELS-related effects.

## Introduction

MicroRNAs (miRNAs) represent one of the major small non-coding RNA classes that have been proposed as regulatory molecules in several biological processes, including neurodevelopment and stress response (1–4). They are critical regulators of gene expression and exert their activity through the modulation of target mRNA stability or translation efficiency. Indeed, the miRNA binding, primarily to the 3′UTR of mRNAs, leads to mRNA destabilization or translational repression, resulting in reduced protein levels of the miRNA-target genes (5). Therefore, miRNAs have been also named as “master regulators”, because one miRNA can regulate hundreds of genes within a specific biological or cellular pathway and more than half of the protein-coding genes are predicted to be regulated by miRNAs (6).

Alterations in miRNAs profiles have been implicated in several psychiatric and neurodevelopmental disorders, as Major Depressive Disorder (MDD) (7–9) and Schizophrenia (SZ) (10–13), two complex and severe diseases characterized by dysregulation of behavior, emotion, and cognition (14). Although the potential role of epigenetic deregulation in the pathogenesis of psychiatric disorders is a major focus of the current research (15, 16), the contribution of individual miRNAs in brain development and function and, consequently, in the pathophysiology of psychiatric illnesses is still largely unknown.

Epidemiological and experimental findings indicate that early life adversities occurring during the pre, peri and postnatal period can increase the vulnerability of developing psychiatric disorders later in life (17–19). Indeed, different environmental factors, including exposure to maternal stress, psychiatric disorders, alcohol, drugs, chemicals, poor nutrition, and infections during pregnancy have a negative impact on brain development during fetal and postnatal life and they have been indeed associated with the future onset of psychiatric disorders or altered behaviours in the offspring (20). Moreover, childhood trauma is a well-known clinical risk factor for the development of psychopathology later in life in the exposed individuals (21–23). For instance, several meta-analyses have provided robust evidence for an association between childhood trauma and SZ (24, 25). For example, a longitudinal 10-year prospective cohort study of 3,021 adolescents and young adults showed that experiences of childhood trauma and recent life events (namely, the second challenge) are strongly correlated and interact additively in increasing the risk for psychosis (26). Similarly, in another prospective study, a large cohort of adolescents and adults who have been sexually abused before the age of 16 years old showed a 2-fold increased risk for a psychotic disorder and a 2.6-fold increased risk for SZ (27).

Recent studies have identified miR-19 as a key regulator of neurodevelopmental brain trajectories, since it drives the differentiation of neural stem cells into mature neurons (28–31). MiR-19, a family composed of miR-19a and miR-19b that differ from each other by one single nucleotide in the middle of the sequence is a member of a polycistronic miRNA gene, namely, the miR-17/92 cluster. This cluster includes miR-17, miR-18a, miR-19a, miR-20a, miR-19b, and miR-92a and is mainly involved in brain development and function. In humans, a microdeletion of this cluster results in the Feingold syndrome type II characterized by microcephaly, mild intellectual disabilities, and psychiatric symptoms, including MDD and anxiety (32–34). Conversely, a microduplication of the genomic locus including miR-17/92 results in mild macrocephaly and autism spectrum disorder (35). Several studies have also established that the miR-17/92 cluster regulates the proliferation and oligodendrogenesis of neural progenitor cells (NPCs) during brain development (30, 36, 37). In this context, Bian and collaborators have recently demonstrated that miR-19 exerts a key role in the developing mouse neocortex, promoting neural stem cell proliferation, and modulating the NPCs fate, through the repression of phosphatase and tensin homolog (PTEN) protein (30). Similarly, Han and collaborators have revealed that an over-expression of miR-19 in adult hippocampal NPCs facilitates cell migration and newborn neuron deposition in the adult brain by directly targeting Rap Guanine Nucleotide Exchange Factor 2 (RAPGEF2), a member of the Rapgef family known to be involved in neuronal migration (38). These data confirm the involvement of miR-19 in neurodevelopment, above all in the regulation of the first steps of adult neurogenesis. Moreover, by using human hippocampal NPCs derived from adult SZ patients (SZ-NPCs) and relative controls, the same authors (29) found that miR-19 expression levels were higher in SZ-NPCs as compared to those obtained from controls. This finding is consistent with a previous study reporting increased levels of miR-19 in post-mortem brains of SZ patients (39) and suggests this miRNA as a molecule associated to SZ, with the potential to affect brain connections and functions.

Although the above-mentioned studies have demonstrated that alterations in miR-19 expression are associated with an aberrant neurodevelopment or with a specific clinical feature, none of these studies has focused the attention on the effects of early life stress (ELS), one of the most important clinical risk factor for mental disorders, which negatively affects brain developmental trajectories, in modulating miR-19 levels.

Therefore, considering that stressful experiences in sensitive periods of life, when brain is still under maturation, can influence the correct processes of neurodevelopment (40–43) as well as neurogenesis (44), in this study, we aimed to investigate whether ELS can modulate miR-19 expression levels and its related pathways and whether its long-lasting effects persisted overtime. In our previous paper (4), we performed miRNomics analyses in different tissues and species all mimicking a condition of early stress, and, among the significantly modulated miRNAs, we identified miR-19a-3p, which was downregulated in proliferating HPCs treated with cortisol, and miR-19b-3p, which was downregulated as well in the hippocampus of adult prenatal stress (PNS) exposed rats as compared to control animals. Based on these findings, in the current work, we decided to follow up our results on this miRNA, examining more in details the short and long-lasting effects of ELS on miR-19 expression levels and also investigating the miR-19 variants represented by miR-19a and miR-19b. Moreover, we have also measured a panel of miR-19 validated target genes involved in neurodevelopment and inflammation, to evaluate the role of miR-19 in the modulation of these biological pathways.

We tested our hypothesis by measuring the short and long-term modulation of miR-19a and miR-19b in our well-established *in vitro* model of stress represented by HPCs treated with cortisol, the stress hormone, during 3 days of proliferation or after 20 days of differentiation into mature neurons without any treatment. We also measured miR-19a and miR-19b in the hippocampus of adult rats exposed *in utero* to PNS as compared to control animals.

## Materials and Methods

### Cell Culture

The immortalized multipotent, human hippocampal progenitor cell line, HPC0A07/03C (propriety of ReNeuron), was used for all the experiments. Because of these cells are grown in a tightly controlled experimental environment, this model allows overcoming the unavoidable variability of clinical samples and to reproduce data from human brain cells, inaccessible in patients (44). As previously described (4, 45), HPC0A07/03C cells proliferate indefinitely in the presence of epidermal growth factor (EGF), fibroblast growth factor (bFGF), and 4-hydroxytamoxifen (4-OHT), whereas differentiation is induced by the removal of these molecules.

We performed two different treatments. **Treatment 1 (short-term effects):** HPC0A07/03C cells were treated for 3 days during proliferation with cortisol (100 µM) or vehicle and cells were harvested immediately after the end of the treatment. **Treatment 2 (long-term effects):** HPC0A07/03C cells were treated with cortisol (100 µM) or vehicle during 3 days of proliferation and harvested after 20 days of differentiation into mature neurons without any treatment. Five biological replicates were obtained for each treatment and condition (cortisol or vehicle).

By using these *in vitro* paradigms, we aimed to identify changes in miR-19 expression levels as consequence of the direct exposure to stress during development (mimicked by cortisol treatment in the proliferation phase, namely, Treatment 1) and to evaluate whether such changes could be maintained over time, after differentiation into mature neurons (Treatment 2).

### PNS Model

The PNS paradigm was performed as already published (4, 40, 46). Briefly, pregnant dams were restrained in a transparent Plexiglas cylinder, under bright light, for 45 min, three times a day during their last week of gestation. Control pregnant females were left undisturbed in their home cages. Adult male offspring from the control and the PNS groups was sacrificed at postnatal day (PND) 62 for the whole dissection of the hippocampus. Rat handling and experimental procedures were performed according to the EC guidelines (EC Council Directive 86/609 1987) and with the Italian legislation on animal experimentation (D.L. 116/92), in accordance with the National Institute of Health Guide for the Care and Use of Laboratory Animals. RNA samples from the hippocampus of animals exposed or not to PNS (n = 10 per group) at PND 62 were used for the expression analyses of miR-19a, miR-19b, and its target genes.

### RNA Isolation

Total RNA, including miRNAs, was isolated from HPC0A07/03C cells using the AllPrep DNA/RNA/miRNA kit (Qiagen, Hilden, Germany) and from rats’ brains using PureZol RNA isolation reagents (Bio-Rad Laboratories, Hercules, CA, USA), according to the manufacturer’s protocols. RNA quantity and quality were assessed by evaluation of the A260/280 and A260/230 ratios using a Nanodrop spectrophotometer (NanoDrop Technologies, Wilmington, DE, USA).

### Real-Time PCR

The expression levels of miR-19 were analyzed in proliferating or differentiated HPC0A07/03C cells (treated with cortisol 100 μM or vehicle) by Real-Time PCR using the CFX384 instrument (Bio-Rad Laboratories, Hercules, CA, USA) and the TaqMan MicroRNA Assays (Thermofisher, Waltham, MA, USA), following the manufacturer’s instructions.

MiR-19 includes miR-19a and miR-19b, which differ from each other by one single nucleotide in the middle of the sequence, so we measured both of them in our experimental model (for details, see https://www.thermofisher.com/order/genome-database/details/mirna/000395 and https://www.thermofisher.com/order/genome-database/details/mirna/000396). In order to analyze the modulation of miR-19a and miR-19b, a total amount of 50 ng of the extracted total RNA, including miRNAs, from each sample was firstly reverse transcribed using the TaqMan MicroRNA RT Kit (Thermofisher, Waltham, MA, USA) and, subsequently, the RT product was pre-amplificated using TaqMan PreAmp Master Mix (Thermofisher, Waltham, MA, US). At this point, the PreAmp product was diluted with 0.1X TE and then evaluated for the expression levels analysis of miR-19a and miR-19b by Real-Time PCR using the CFX384 instrument (Bio-Rad Laboratories, Hercules, CA, USA), the TaqMan MicroRNA Assays (Thermofisher, Waltham, MA, USA) and the TaqMan Universal Master Mix, no AmpErase UNG (Thermofisher, Waltham, MA, USA) following the manufacturer’s instructions. The relative expression of miR-19a and miR-19b was normalized to the levels of the housekeeping gene RNU44 in HPC0A07/03C cells (47, 48) and to the levels of U6 in rats. All the reactions were performed in triplicates.

The expression levels of several miR-19 validated target genes, such as Phosphatase And Tensin Homolog (PTEN), Phosphoinositide-3-Kinase Regulatory Subunit 1 (PIK3R1), Adrenoceptor Beta 1 (ADRB1), Neuronal Cell Adhesion Molecule (NRCAM), Transforming Growth Factor Beta Receptor 2 (TGFBR2), RAPGEF2, Tumor Necrosis Factor alpha (TNFα), Interleukin 4 Receptor (IL4R), and the housekeeping gene Glyceraldehyde-3-Phosphate Dehydrogenase (GAPDH) were evaluated in the hippocampus of adult PNS rats by using TaqMan Assays (Thermofisher, Waltham, MA, USA) on the CFX384 instrument (Bio-Rad Laboratories, Hercules, CA, USA), following the manufacturer’s instructions.

The Pfaffl Method was used to determine the relative expression values of miR-19a, miR-19b and of genes of interest (49).

### Gene-Targeting Prediction and Validation Analyses

The gene-targeting analyses for predicted and validated target genes of miR-19a and miR-19b were performed by using miRWalk2.0 database (http://zmf.umm.uni-heidelberg.de/apps/zmf/mirwalk2/) (50), a comprehensive database that provides predicted as well as validated miRNA binding site information. To make any possible inference and to obtain a comparative view, miRWalk automatically combines and integrates the identified miRNA binding sites with the results obtained from other established miRNA-target prediction databases (miRWalk, miRanda, and Targetscan). Specifically, we used the sequences, annotation and accession number available in the miRBase database (http://www.mirbase.org/) (51), such as MI0000073 for mir-19a, MI0000074 for mir-19b-1, and MI0000075 for mir-19b-2.

### In Silico Analysis of Candidate Genes

In order to evaluate the potential involvement of differentially regulated genes in association with the effects of exposure to stress early in life, we considered open target genetic prioritization platform (https://genetics.opentargets.org/). This platform performs an integrative analysis including the evaluation of genetic signal from genome-wide association study (GWAS) Catalog and UK Biobank as well as databases annotation from multiplex public available resources (genetic association, expression, literature, animal models). By integrating different kind of data from omics data to automatic information retrieval from literature, the open target platform is capable to visualize target gene-disease associations (52). The combination of GWAS and functional genomics data have allowed to prioritise likely causal variants at disease-associated loci (53).

Moreover, we performed a Transcriptome Wide Association Study (TWAS) for MDD and SZ in human considering brain tissues and blood (http://twas-hub.org/). These models integrate genome wide associations for a given phenotype (i.e., MDD or SZ) with expression quantitative loci (eQTL) from gene-expression tissue database, such as GTEx (https://gtexportal.org/home/) and common mind consortium (https://www.nimhgenetics.org/resources/commonmind) to test whether or not a differential tissue-specific gene expression regulation can be expected according to the genetic and the eQTL signal. By mean of TWAS, it is possible to prioritize gene associations starting from GWAS by estimating gene-expression downregulation and upregulation in a tissue specific manner. In fact, many genetics variants influence complex trait by modulating gene expression. Specifically, TWAS quantify the association between the genetically regulated levels of gene expression and the phenotype by mean of different regression models (54).

### Pathway and Network Analyses

Lists of validated target genes of miR-19a, miR-19b-1, and miR-19b-2 were imported into Ingenuity Pathway Analyses Software (IPA) for pathway and network analyses. The “Core Analysis” function included in IPA (Ingenuity System Inc., USA http://www.ingenuity.com) was used to understand and discuss the data in the context of biological processes, pathways and networks associated with the experimental system. Kyoto Encyclopedia of Genes and Genomes (KEGG) pathway database (https://www.genome.jp/kegg/pathway.html) was used to investigate the molecular interaction, reaction, and relation networks among the biological systems.

### Statistical Analyses

Statistical analyses were conducted using SPSS version 24.0 statistical software (SPSS Inc., Chicago, IL, USA) and data are expressed using box-plot. For comparison of variables between groups, Student’s t-test was applied.

## Results

### Effect of ELS on miR-19a and miR-19b Expression Levels in the In Vitro Model

**Figure 1:**
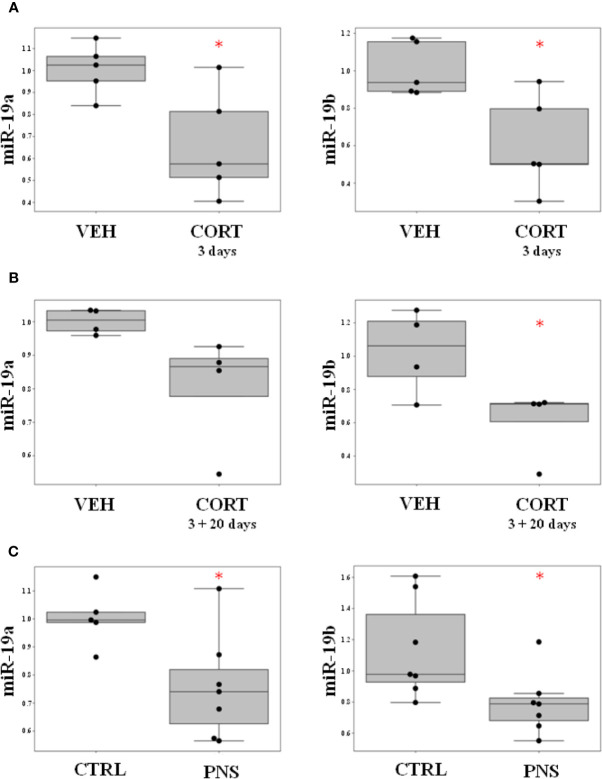
**(A)** Effect of ELS on the modulation of miR-19a and miR-19b in HPC0A07/03C cells treated with cortisol during 3 days of proliferation as compared to cells treated with vehicle. **(B)** Effect of ELS on the modulation of miR-19a and miR-19b in HPC0A07/03C cells treated with cortisol during 3 days of proliferation and differentiated into mature neurons for 20 days as compared to cells that received vehicle. **(C)** Effect of PNS on the modulation of miR-19a and miR-19b in adult PNS rats (PND 62) as compared to control animals. Data are shown using boxplot, *p-value < 0.05.

With the aim to investigate the effects of ELS on the modulation of miR-19a and miR-19b, we used our well-established *in vitro* model of stress represented by the HPC0A07/03C cells that we treated with the stress hormone cortisol 100 μM (20, 40, 45). First, we looked at miR-19a and miR-19b expression levels immediately after 3 days of cortisol treatment and we observed a statistically significant down-regulation of both miRNAs in proliferating cells as compared to vehicle (FC = −1.59, p-value = 0.022 for miR-19a; FC = −1.79, p-value = 0.016 for miR-19b) (**Figure 1A**). Interestingly, after 20 days of differentiation without any treatment, we found a similar trend of decrease of miR-19a (FC = −1.28, p-value = 0.065) and a significant downregulation of miR-19b expression levels (FC = −1.75, p-value = 0.047) in mature neurons, which were previously sensitized by cortisol treatment during 3 days of proliferation as compared to vehicle (**Figure 1B**).

These *in vitro* results suggest that an exposure to high concentration of cortisol affects the short-term modulation of miR-19a and miR-19b with long-lasting effects that can be also observed after 20 days of cells differentiation.

### Biological Systems Regulated by miR-19 Validated Target Genes

As we were interested in investigating the biological processes and signaling targeted by miR-19a and miR-19b, we performed gene-targeting analyses using miRWalk2.0 database. Specifically, we looked at predicted as well as validated target genes of miR-19a, miR-19b-1, and miR-19b-2. We obtained lists of thousands of genes as predicted targets of all the three miRNAs (data not shown), a list of 631 validated target genes for miR-19a, a list of 787 validated target genes for miR-19b-1, and a list of 780 validated target genes for miR-19b-2 (see **Supplementary Tables 1****–****3**). Thus, in order to restrict the datasets and to reduce the number of genes to focus on, we only considered the validated target genes.

**Figure 2:**
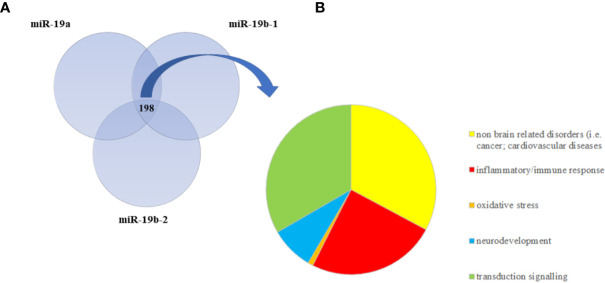
**(A)** The Venn diagram represents the overlap between statistically significant pathways potentially regulated by validated target genes of miR-19a, miR-19b-1, and miR-19b-2. The intersection refers to 198 common significant pathways, which are mainly involved in inflammation, neurodevelopment, and intracellular signal transduction. **(B)** The pie chart summarizes the relevant biological functions of all 198 common significant pathways.

We then performed a pathway analysis on each of these three lists of validated target genes by using IPA. We identified 229 statistically significant pathways potentially targeted by miR-19a, 220 potentially regulated by miR-19b-1, and 210 by miR-19b-2 that we listed in **Supplementary Tables 4****–****6**. In order to only select common pathways regulated by all the three miRNAs, we overlapped the three lists of pathways by using Venn diagram and we found 198 common significant pathways (**Figure 2A** and **Supplementary Table 7**), including, as the most representative ones, those involved in the *inflammatory and immune response* (i.e., TGFβ signaling, IL-6 signaling, NF-KB signaling, B cell receptor signaling, IL-2 signaling, T cell receptor signaling, Chemokine signaling), in *neurodevelopment* (i.e., Role of NANOG in Mammalian Embryonic Stem Cell Pluripotency, NGF Signaling, P2Y Purigenic Receptor Signaling Pathway, Neuregulin Signaling, Synaptic Long Term potentiation, HIPPO signaling), as well as in the *intracellular signal transduction* (i.e., JAK/STAT signaling, ERK/MAPK signaling, ErbB signaling, PI3K/AKT signaling, AMPK signaling, RAC signaling, RhoA signaling). We also graphically represented all the significant common pathways in a pie chart detailing the relevant biological and functional processes (**Figure 2B**). Interestingly, among the most significant common pathways, we also found the Glucocorticoid Receptor Signaling (**Supplementary Table 7**), confirming the key role of miR-19 in stress response.

### Bioinformatics Analyses of miR-19

Integrative analyses on open target platform (https://www.targetvalidation.org/) revealed a potential association of miR-19 target genes with MDD and SZ. Noteworthy, TNF60, PIK3R1, TP53, PINK1, NRCAM, IL4R, ADRB1, PTEN, and RAPGEF2 show an association with SZ at different level of evidence (genetic association, expression, and literature) and, interestingly, IL4R and ADRB1 have been found also associated with MDD. Moreover, TWAS analysis in SZ considering candidates gene from inflammatory and neurodevelopmental pathways suggest that genetic component of SZ [GWAS signal from (55)] is associated with the gene expression regulation of pivotal genes involved in these pathways (**Table 1**). Specifically, among the validated target genes, we selected TGFBR2, TNFα, IL4R, ADRB1, NRCAM, and PIK3R1 taking into account their involvement in pathways related to inflammatory and immune response (i.e., TGFBR2, TNFα, IL4R) and neurodevelopment (i.e., ADRB1, NRCAM, PIK3R1). We also selected PTEN and RAPGEF2 according to their well-established interaction with miR-19, as suggested by literature data (29, 30).

**Table 1:** Schizophrenia TWAS association of the analysed inflammatory and neurodevelopmental genes in blood and brain tissues.

Tissue	Gene	Z-score	p-value	Process
Brain Cerebellum	SLC25A12	4.27	1.93E-05	Neurodevelopment
Brain Cortex	SLC25A12	3.81	1.40E-04	Neurodevelopment
Brain Hippocampus	SLC25A12	3.42	6.23E-04	Neurodevelopment
Brain Cerebellar Hemisphere	TNIP1	−2.69	7.20E-03	Inflammation
Cerebellar Hemisphere	MAP3K9	2.61	9.18E-03	Inflammation
Whole Blood	DUTP6	2.50	1.25E-02	Neurodevelopment
Brain Hippocampus	STK4	2.41	1.59E-02	Inflammation
Brain Caudate basal ganglia	SNX27	2.37	1.79E-02	Neurodevelopment
Brain Caudate basal ganglia	DUTP6	2.29	2.18E-02	Neurodevelopment
Brain Nucleus accumbens basal_ganglia	FAS	2.18	2.95E-02	Inflammation
Brain Hippocampus	STK4	2.16	3.04E-02	Inflammation
Brain Frontal Cortex	RPA2	2.15	3.15E-02	Neurodevelopment
Whole Blood	RPA2	1.99	4.67E-02	Neurodevelopment
Brain Nucleus accumbens basal ganglia	DUTP6	1.98	4.75E-02	Neurodevelopment
Brain Cerebellum	LRP8	1.97	4.84E-02	Neurodevelopment

Only significant (p-value < 0.05) genes are reported in the table. Z-score represents the directionality of gene expression regulation (i.e., down- or upregulation).

**Figure 3:**
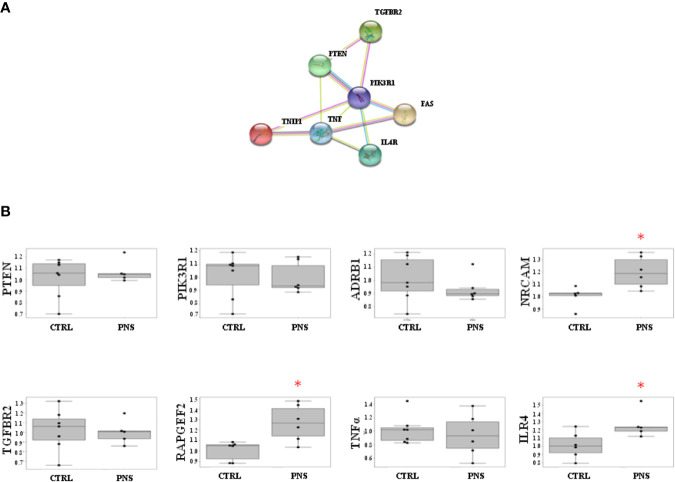
**(A)** STRING protein-protein interactions between validated target genes of miR-19 selected for Real-Time PCR analyses. The protein-protein interaction analysis shows an enrichment of interactions with TWAS genes involved in neurodevelopmental and inflammatory processes (p-value = 0.67E-04), and is corroborated by the presence of a sub-cluster of such genes with at multiple levels of interactions evidences. **(B)** Effects of PNS on validated target genes of miR-19a and miR-19b in adult PNS rats (PND 62) as compared to control animals. PNS significantly upregulates the expression levels of NRCAM, IL4R, and RAPGEF2. Data are shown using boxplot; *p < 0.05.

Noteworthy, the analysis of STRING interaction databases suggests an enrichment of interactions between the analysed genes and inflammatory/neurodevelopmental TWAS significant genes. In particular, the protein-protein interaction analysis shows an enrichment of interactions with TWAS genes involved in neurodevelopmental and inflammatory processes (p-value = 0.67E-04). This is further corroborated by the presence of a sub-cluster of such genes with at multiple levels of interactions evidences (see **Figure 3A**).

### Effect of PNS on miR-19a and miR-19b Expression Levels in the Adult Offspring

In order to corroborate that stress early in life could have a long-term effect on the modulation of both miR-19a and miR-19b, we measured both miRNAs expression levels in the hippocampus of adult male rats (PND 62) whose mothers were exposed to PNS during the last week of gestation as compared to controls.

Interestingly, we observed a statistically significant reduction in the expression levels of both miR-19a and miR-19b in the adult PNS offspring as compared to controls (FC = −1.35, p-value = 0.025 for miR-19a; FC = −1.43, p-value = 0.032 for miR-19b) (see **Figure 1C**).

### Expression Levels of miR-19 Validated Target Genes in the Hippocampus of Adult PNS Rats

In order to investigate the modulation of miR-19 validated target genes, we have analyzed the mRNA levels of PTEN, PIK3R1, ADRB1, NRCAM, TGFBR2, RAPGEF2, TNFα, and IL4R in the hippocampus of adult PNS rats (PND 62) as compared to control animals. Specifically, we selected these validated target genes taking into account their involvement in pathways related to *inflammatory and immune response* (i.e., TGFBR2, TNFα, IL4R) and *neurodevelopment* (i.e., ADRB1, NRCAM, PIK3R1). We also selected PTEN and RAPGEF2 according to their well-established interaction with miR-19, as suggested by literature data (29, 30). Interestingly, among all the analyzed genes, we observed a significant increase of NRCAM, IL4R, and RAPGEF2 in the adult PNS rats as compared to controls (FC = 1.20, p-value = 0.027 for NRCAM; FC = 1.26, p-value = 0.046 for IL4R; FC = 1.23, p-value = 0.020 for RAPGEF2) (**Figure 3B**).

**Figure 4:**
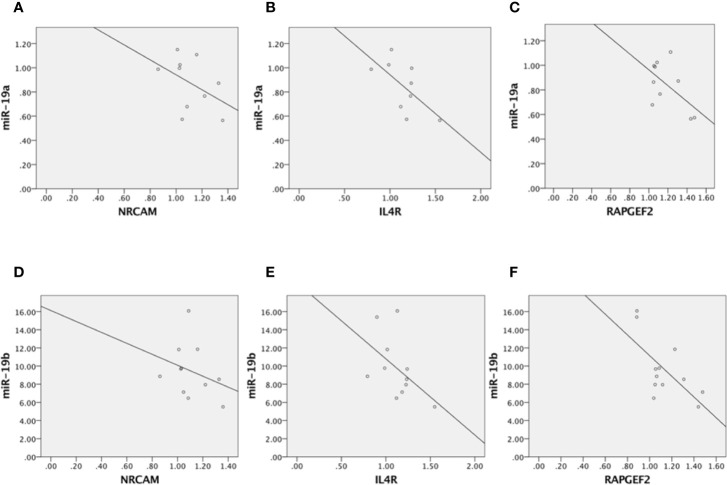
Correlation analyses between miR-19a and NRCAM **(A)**, miR-19a and ILR4 **(B)**, miR-19a and RAPGEF2 **(C)**, miR-19b and NRCAM **(D)**, miR-19b and ILR4 **(E)**, and miR-19b and RAPGEF2 **(F)**.

In addition, we performed correlation analyses between the modulation of miR-19a and miR-19b and the expression levels of these target genes in PNS rats and control animals, in order to identify potential associations between miR-19 and its targets. Overall, we observed inverse correlations between the mRNA levels of all the three genes and the levels of miR-19a and miR-19b, that however reached the significance only for RAPGEF2 and miR-19b (p-value = 0.019, r2 = 0.438). In addition, a trend of significance was observed between RAPGEF2 and miR-19a (p-value = 0.09, r2 = 0.318) and between ILR4 and miR-19a (p-value = 0.064, r2 = 0.409) (see **Figure 4**). However, these correlation analyses are not able to prove any causality, and further studies based on the use of functional constructs to block the effects of miR-19 on its targets will be helpful to prove the causative role of miR-19 in mediating the effects of ELS on biological processes and more importantly in shaping the future vulnerability of developing a specific phenotype of vulnerability.

## Discussion

In the present study, we observed that an exposure to ELS produces a short and long-term effect on miR-19 modulation, which in turn may affect normal trajectories of brain development and neuronal networks formation, increasing the vulnerability to develop psychiatric disorders later in life.

According to a growing body of evidence, miR-19 expression is required for a proper neurodevelopment, as it controls the expansion of neural stem cells and radial glial cells, which are essential for normal cortical development and function (30). In addition, miR-19 has been suggested to be a key regulator of hippocampal neurogenesis (29, 38).

In human beings, the immature brain is highly plastic but particularly vulnerable to a range of environmental insults, including ELS, which can result in long-term cognitive and behavioral impairment (56). Indeed, a wide number of stressful environmental factors occurring during sensitive periods of life, such as maternal exposure to bereavement, natural disasters or terrorism as well as financial and relationship problems in pregnancy (57), or exposure to traumatic events in childhood (58), can compromise normal trajectories of brain development and neuronal networks formation, leading to abnormal programming of brain circuits (59).

Although the contribution of epigenetic mechanisms, including miRNAs, has been widely demonstrated in mediating the effects of ELS on brain functions (60, 61), to our knowledge, no studies have so far investigated the effects of ELS on miR-19 expression levels. Interestingly, in our previous paper (4), among the significantly modulated miRNAs identified through a miRNomics approach, we found miR-19a-3p as significantly downregulated in proliferating HPCs treated with cortisol and miR-19b-3p as significantly downregulated in the hippocampus of adult PNS exposed rats as compared to control animals. Based on these data, in this paper, we decided to follow up our results on miR-19 and to examine more in depth both the short- and long-lasting effects of ELS on the expression levels of this miRNA, the modulation of its variants and the involvement of possible biological signaling affected by miR-19 in association to ELS exposure.

Therefore, in this study, we first analysed the expression levels of miR-19a and miR-19b in our “*in vitro*” model of stress represented by HPC0A07/01C cells treated with high concentration of cortisol, a condition that we have previously found associated with a reduced neurogenesis. We observed that the levels of miR-19a and miR-19b were downregulated in proliferating cells treated with cortisol for 3 days as compared to vehicle. Moreover, the same effect was observed also in mature neurons, previously sensitized by cortisol, after 20 days of differentiation without any treatment. These data suggest that the expression levels of miR-19a and miR-19b begin to be modulated by cortisol during proliferation but persist overtime.

A similar result has been recently found by Provencal and collaborators, who reported an association between the exposure of HPCs to glucocorticoids at different stages (during proliferation and differentiation, but not after differentiation) and long-lasting changes in mRNA expression and DNA methylation profiles. Specifically, the long-lasting alterations in DNA methylation correlated with an enhanced responsivity to a second glucocorticoid challenge later in life (62), suggesting that early exposure to glucocorticoids may have a long-lasting impact on the development of the nervous system by priming the global expression levels of relevant genes. These findings suggest that prenatal exposure to glucocorticoids could not only alter neurodevelopmental trajectories, but also change the set point of stress reactivity of adult tissues, increasing the risk for stress-related psychiatric disorders.

These data corroborate the so called “double hit” hypothesis, suggesting that ELS may act as a first negative “hit”, predisposing individuals to be more vulnerable to subsequent negative stressful challenges, namely, the “second hit”, later in life (63–65).

To further support our results in the *in vitro* model, we took advantage of a well-established animal model of PNS and we measured the expression levels of both miR-19a and miR-19b in the hippocampus of adult PNS male rats (PND 62) as compared to their controls, reporting a downregulation of both the miRNAs.

In line with our data, several studies have demonstrated that the exposure to prenatal or to early postnatal stress produces long-lasting alterations in the expression of molecules involved in biological systems important for brain plasticity. For instance, it is well known that rodents exposed to PNS show reduced expression levels of brain-derived neurotrophic factor (BDNF), a marker of neuronal plasticity (66). Accordingly, in a recent study performed by our group (67), a significant disruption in the novel object recognition test was found both in male and female adult rats exposed to PNS, although such impairment was more pronounced in females. The cognitive dysfunction observed during the behavioral test in adult PNS animals appeared to be the consequence of an abnormal activation of a pattern of genes, which was required for proper cognitive performances. Moreover, functional alterations originating from PNS exposure could represent the consequence of an inability to activate the proper transcriptional machinery required for the correct cognitive performance in relevant brain areas, such as the dorsal hippocampus. All these findings suggest that an exposure to stress early in life represents a priming event that interferes with the correct and physiological response during a cognitive task (67). Therefore, we suggest that the exposure to ELS not only influences the development of brain trajectories as well as the maturation of specific brain networks, but also affects cognitive impairments.

Although in the present study, PNS and control rats were not assessed for behavioural tests, previous studies also coming from our group have demonstrated that an exposure to PNS is associated with altered behavior in the offspring, both in adulthood (67, 68) as well as at peri-adolescence (66). Thus, the presence of lower levels of both miR-19a and miR-19b levels in the hippocampus of animals exposed to PNS could represent a biological signature of vulnerability that could shape the individual risk of developing altered behaviors and cognitive deficits later in life.

In order to investigate whether the biological processes regulated by miR-19a and miR-19b could be affected by ELS, we first performed gene-targeting analyses focusing only on validated target genes and subsequently we ran pathway analyses on these subsets of genes. We found that the most significant pathways modulated by miR-19a and miR-19b were involved in *neurodevelopment*, as we expected, but also in the *inflammatory/immune response* and in the *intracellular signal transduction*. In line with our results, a growing body of evidence has confirmed that ELS may alter the neuroinflammatory processes that interact with brain development, leading to a sensitized immune response and heightened neuroinflammation later in life, enhancing the risk for psychiatric disorders (69, 70). Accordingly, several clinical studies have demonstrated that ELS, both prenatally and in childhood, is associated with an increased inflammatory state, in terms of high C-reactive protein (CRP) and pro-inflammatory cytokines levels, later in life (71). This has been clearly described in the prospective cohort study performed by Baldwin and colleagues (72), who reported an association between childhood victimization, elevated levels of CRP at the age of 18 and the development of psychopathology later on. Similarly, a recent and interesting meta-analysis has clearly supported the association between the exposure to different types of childhood trauma and the presence of a pro-inflammatory state, represented by high CRP, Interleukin (IL)-6, and Tumor Necrosis Factor (TNF)-α levels (73).

To prove that the modulation of miR-19 could affect the long-term expression levels of pathways related to brain functions, stress response, and inflammation, we specifically analysed, in the hippocampus of adult rats exposed or not to PNS, the mRNA levels of a panel of genes targeted by miR-19 and involved in these signalling. Among all these validated target genes, we found increased expression levels of NRCAM, IL4R, and RAPGEF2 in PNS rats as compared to control animals at early adulthood, in line with a significant decrease in the mRNA levels of both miR-19 a and miR-19b observed in the same animals, as already described.

NRCAM, a member of the immunoglobulin superfamily, is involved in neural development including cell proliferation and differentiation, axon growth and guidance, as well as synapse formation. In the last years, genetic association studies have shown that alterations in NRCAM are associated with psychiatric disorders, such as SZ, autism, and drug addiction (74). Since NRCAM is involved in key brain functions and psychiatric disorders can be caused by the disruption of any of these processes, we can speculate that changes in the expression levels of this gene, possibly due to the modulation of miR-19, may introduce subtle but significant effects on brain development and wiring, which by themselves might be sufficient to increase the risk for psychopathologies.

*Interleukin 4 receptor* (IL4R) is a transmembrane protein that plays a critical role in binding IL4, a cytokine involved in several biological processes. Indeed IL4R not only regulates immune functions, such as IL4-mediated IgE production and Th2 inflammatory reactions (75), but it plays also a key role in pregnancy, fetal development, as well as in higher brain functions including memory and learning (76). Recently, IL4R has been also suggested to be directly involved in the promotion of neuronal survival and sprouting (77), thus preserving cognitive functions (77). Although IL4R is necessary for normal brain development (75), many aspects of neural IL4R expression, regulation, and signalling in both brain repair and disease remain to be examined.

By evaluating the association between fetal inflammation and neurodevelopmental delay at age 2, Clarke and collaborators have suggested that genetic alterations to the extracellular domain of IL4R may contribute to neurodevelopmental outcomes in pregnancies at high risk for preterm birth (78).

On these bases, we can hypothesize that changes in the expression levels of IL4R, following an exposure to ELS and possibly regulated by miR-19, may disrupt correct brain development and functions.

Finally, RAPGEF2 governs cell migration by modifying the activity of Rap proteins (79). As widely demonstrated, miR-19 drives cell migration by regulating RAPGEF2 expression levels in adult NPCs and several studies have shown an inverse correlation between the levels of miR-19 and of the target gene. Indeed, in physiological conditions, increased miR-19 levels suppress RAPGEF2 expression by binding to RAPGEF2 mRNA. Consistently with these findings, the migration efficiency of newborn neurons is increased when miR-19 is overexpressed and RAPGEF2 is depleted (Han and Gage, 2016). Based on this evidence, our results, showing that ELS decreases miR-19a and miR-19b expression levels already after 3 days of cell proliferation, and also after differentiation, might suggest that the presence of lower levels of miR-19 may not be able to suppress RAPGEF2 activity during the first steps of neural development, such as hippocampal neurogenesis. This could thus contribute in causing impairments in brain maturation and in rendering the system more vulnerable for the development of neurodevelopmental and stress-related disorders.

In conclusion, our data suggest that alterations in miR-19 levels as consequence of ELS exposure could cause alterations in pathways involved in neurodevelopment and in the immune system. Intervention strategies targeting miR-19 might be useful to prevent alterations in these pathways and to reduce the ELS-related effects. Of note, in our previous paper, we found a downregulation of miR-125b-1-3p in the same models used in this study and in particular in HPCs treated with high concentration of cortisol and in the hippocampus of PNS rats. Looking more in details at the possible biological systems modulated by target genes of these miRNAs, interestingly, we have observed that both miR-125b-1-3p and miR-19 are able to regulate pathways involved in the inflammatory and immune response and also in the neurodevelopment. Specifically, among the significant pathways regulated by both miR-125b-1-3p and miR-19, there are several pathways associated with inflammation and immune response, such as TGFβ signalling, CCR3 signalling in eosinophils, fMLP signalling in neutrophils, role of NFAT in regulation of the immune response. Only one common pathway, P2Y Purigenic Receptor Signalling Pathways, is more related to neurodevelopment. Altogether, these findings suggest that exposure to stress early in life can act on the modulation of several miRNAs, including miR-125b-1-3p and miR-19, which can play crucial roles in biological processes and functions involved in inflammation and immune response. These miRNAs can represent a possible epigenetic mechanism underlying the long-lasting effect of ELS that shape the individual vulnerability to develop stress-related psychiatric disorders later in life. However, further studies are needed to better dissect the contribution of these miRNAs and their common target pathways in association with ELS exposures.

## Data Availability Statement

The datasets generated for this study are available on request to the corresponding author.

## Ethics Statement

The animal study was reviewed and approved by Italian legislation on animal experimentation (D.L. 116/92).

## Author Contributions

MM, CrM, NM, VB, and NC contributed to the experiments in the *in vitro* and *in vivo* model. MM and NC managed the literature searches. MM, CaM, and NC performed the bioinformatics and statistical analyses. MM and NC contributed to the first draft of the manuscript, CP, MR, and AC contributed to the revision, and NC revised all the versions of the manuscript and approved the final one. All the authors have contributed to and have approved the final manuscript.

## Funding

Funding from the Italian Ministry of Health (MoH) (Ricerca Corrente) to AC and NC supported this work.

## Conflict of Interest

The authors declare that the research was conducted in the absence of any commercial or financial relationships that could be construed as a potential conflict of interest.

## Supplementary Material

Supplementary Table 1 List of 631 validated target genes of miR-19a.

Supplementary Table 2 List of 787 validated target genes of miR-19b-1.

Supplementary Table 3 List of 780 validated target genes of miR-19b-2.

Supplementary Table 4 229 statistically significant pathways regulated by miR-19a.

Supplementary Table 5 220 statistically significant pathways regulated by miR-19b-1.

Supplementary Table 6 210 statistically significant pathways regulated by miR-19b-2.

Supplementary Table 7 List of 198 common significant pathways regulated by miR-19a, miR-19b-1 and miR-19b-2.
