# Corrigendum: The Long-Term Effects of Early Life Stress on the Modulation of miR-19 Levels

**DOI:** 10.3389/fpsyt.2020.643932

**Published:** 2021-02-15

**Authors:** Monica Mazzelli, Carlo Maj, Nicole Mariani, Cristina Mora, Veronica Begni, Carmine M. Pariante, Marco A. Riva, Annamaria Cattaneo, Nadia Cattane

**Affiliations:** ^1^Biological Psychiatry Unit, IRCCS Istituto Centro San Giovanni di Dio Fatebenefratelli, Brescia, Italy; ^2^Institute for Genomic Statistics and Bioinformatics, University Hospital, Bonn, Germany; ^3^Stress, Psychiatry and Immunology Laboratory, Department of Psychological Medicine, Institute of Psychiatry, Psychology and Neuroscience, King's College London, London, United Kingdom; ^4^Department of Pharmacological and Biomolecular Sciences, University of Milan, Milan, Italy

**Keywords:** early life stress, miR-19, brain trajectories, neurodevelopment, inflammation, depression, schizophrenia

In the published article, there was an error regarding the affiliations for Annamaria Cattaneo. Instead of having affiliations **1** and **3** they should only have affiliation 1.

The authors apologize for this error and state that this does not change the scientific conclusions of the article in any way. The original article has been updated.

