# Supplementary material for: The Long-Term Effects of Early Life Stress on the Modulation of miR-19 Levels
Source: Front Psychiatry. 2020 May 15;11:389. doi: 10.3389/fpsyt.2020.00389 (PMC7243913; doi:10.3389/fpsyt.2020.00389)
Supplement: Supplementary Table 1 — List of 631 validated target genes of miR-19a. [file Table_1.docx]

**Supplementary Table 1.**

| ABCA1 |
| --- |
| ABCC12 |
| ABCG8 |
| ABHD14B |
| ABHD17C |
| ABHD5 |
| ACBD5 |
| ACSL4 |
| ACTB |
| ADIPOR2 |
| ADRBK2 |
| ADRM1 |
| ADSS |
| AFF1 |
| AFTPH |
| AGO1 |
| AGO3 |
| AGPAT5 |
| AHDC1 |
| AKAP2 |
| AKT1 |
| ALG1 |
| ALG2 |
| AMMECR1L |
| ANGEL2 |
| ANKIB1 |
| ANKRD12 |
| ANKRD50 |
| AP3B1 |
| AP3S2 |
| ARAP2 |
| ARC |
| ARFGEF1 |
| ARHGAP1 |
| ARHGAP12 |
| ARHGEF26 |
| ARID1A |
| ARIH2 |
| ARL8A |
| ARMC8 |
| ARPP19 |
| ARRDC3 |
| ASNA1 |
| ATG14 |
| ATG16L1 |
| ATG2B |
| ATG5 |
| ATMIN |
| ATP6V0E1 |
| ATP6V1B2 |
| ATPAF1 |
| ATXN1 |
| ATXN7 |
| AZIN1 |
| B4GALT1 |
| BAMBI |
| BCAS2 |
| BCL2L11 |
| BCL3 |
| BCL7A |
| BCL7B |
| BEND3 |
| BLCAP |
| BMP3 |
| BMPR2 |
| BRD9 |
| BRWD1 |
| BRWD3 |
| BTBD7 |
| BTF3L4 |
| BTG1 |
| BZW1 |
| C11orf57 |
| C15orf38 |
| C15orf38-AP3S2 |
| C16orf70 |
| C2orf42 |
| C5orf24 |
| C5orf30 |
| C5orf51 |
| C6orf132 |
| CAB39 |
| CACNB2 |
| CALM1 |
| CALM2 |
| CAMSAP1 |
| CAMSAP2 |
| CAMTA1 |
| CAPRIN2 |
| CASP10 |
| CASZ1 |
| CBX1 |
| CBX5 |
| CBX7 |
| CBY1 |
| CC2D1A |
| CCDC137 |
| CCDC39 |
| CCDC80 |
| CCNA2 |
| CCND1 |
| CCND2 |
| CCNL1 |
| CCNT2 |
| CCSER2 |
| CD164 |
| CD22 |
| CD38 |
| CEP170 |
| CEP350 |
| CEP55 |
| CERCAM |
| CERK |
| CFL2 |
| CHD9 |
| CHEK1 |
| CHEK2 |
| CHERP |
| CIT |
| CLIC4 |
| CLIP1 |
| CLOCK |
| CLVS2 |
| CMBL |
| CNOT4 |
| CNOT6 |
| CNOT7 |
| COG6 |
| COQ10B |
| CORO1C |
| COX10 |
| CREB3L2 |
| CREBL2 |
| CREBRF |
| CUL5 |
| CXCR6 |
| CYP2U1 |
| DAD1 |
| DBN1 |
| DCAF7 |
| DCC |
| DCP2 |
| DCUN1D3 |
| DDX3X |
| DDX3Y |
| DDX6 |
| DEF8 |
| DENND6A |
| DEPDC1 |
| DGKH |
| DHX40 |
| DICER1 |
| DIP2A |
| DLG5 |
| DMD |
| DMXL2 |
| DNMT1 |
| DPYSL2 |
| DSCR3 |
| DSN1 |
| DSTYK |
| DUT |
| DYNC1LI2 |
| E2F8 |
| EDEM1 |
| EDN1 |
| EFR3A |
| EHD1 |
| EIF4A2 |
| EIF4E2 |
| ELL2 |
| ELMOD2 |
| ELOVL5 |
| ENPP4 |
| ENPP5 |
| EOGT |
| EPN2 |
| EPS15 |
| ERBB4 |
| ERCC4 |
| ESD |
| ESR1 |
| ESR2 |
| ESYT1 |
| ETS1 |
| ETV3 |
| EVI5L |
| EXOC7 |
| FAM102A |
| FAM13B |
| FAM161B |
| FAM195A |
| FAM200B |
| FAM210A |
| FAM46C |
| FAM73A |
| FAM73B |
| FAM83D |
| FAM83G |
| FANCF |
| FAS |
| FBLIM1 |
| FBXO10 |
| FBXO28 |
| FBXO48 |
| FBXO8 |
| FGFR1OP |
| FKBP15 |
| FN3KRP |
| FNDC3A |
| FOS |
| FOXP1 |
| FOXQ1 |
| FRMD6 |
| FRS2 |
| FXR1 |
| FYCO1 |
| FZD6 |
| G3BP2 |
| G6PC |
| GAK |
| GATAD2B |
| GDNF |
| GDPD1 |
| GFPT1 |
| GGCX |
| GID4 |
| GIGYF1 |
| GINS1 |
| GIT2 |
| GMFB |
| GNPTAB |
| GPAM |
| GPATCH8 |
| GPR137B |
| GRB10 |
| GRK6 |
| GRSF1 |
| GSKIP |
| GTF2E2 |
| GTF2H5 |
| HADHB |
| HBP1 |
| HDAC4 |
| HECW2 |
| HIC1 |
| HIPK3 |
| HIST2H4A |
| HIST2H4B |
| HMGA2 |
| HMGXB4 |
| HNRNPF |
| HNRNPR |
| HNRNPU |
| HNRNPUL1 |
| HOMER1 |
| HOXA5 |
| HPRT1 |
| IDNK |
| IGF2BP1 |
| IKZF1 |
| IMPAD1 |
| IMPDH1 |
| INO80 |
| ITGA2 |
| ITPR1 |
| JARID2 |
| JAZF1 |
| KAT2B |
| KATNAL1 |
| KCNJ2 |
| KCTD10 |
| KDELR2 |
| KIAA0907 |
| KIAA1468 |
| KIF13A |
| KIF3A |
| KIT |
| KITLG |
| KLF13 |
| KLF7 |
| KLHDC2 |
| KLHL11 |
| KLHL15 |
| KLHL20 |
| KLHL3 |
| KLHL42 |
| KPNA6 |
| LCLAT1 |
| LDLR |
| LIN9 |
| LMLN |
| LONRF1 |
| LZIC |
| MACF1 |
| MALT1 |
| MAP2K3 |
| MAP3K1 |
| MAP3K14 |
| MAP3K3 |
| MAP3K9 |
| MAPK1 |
| MAPK14 |
| MAPK6 |
| MAVS |
| MB21D1 |
| MB21D2 |
| MBD3 |
| MBD4 |
| MBNL1 |
| MBNL2 |
| MBNL3 |
| MCC |
| MDM4 |
| MECP2 |
| MED12L |
| MED21 |
| MEF2A |
| MFF |
| MFSD6 |
| MID1IP1 |
| MIER1 |
| MKL2 |
| MLEC |
| MLLT10 |
| MMAB |
| MMGT1 |
| MOB1B |
| MOB3B |
| MPRIP |
| MRPL17 |
| MSMO1 |
| MTF2 |
| MTHFD1 |
| MTMR12 |
| MTMR6 |
| MTX3 |
| MXD1 |
| MYC |
| MYCN |
| MYO10 |
| NACC1 |
| NACC2 |
| NAPB |
| NDRG1 |
| NF1 |
| NFATC2IP |
| NFIA |
| NFIB |
| NICN1 |
| NIPA1 |
| NOM1 |
| NPEPL1 |
| NPTN |
| NR4A2 |
| NR4A3 |
| NRBF2 |
| NRBP1 |
| NUFIP2 |
| NUP54 |
| NUS1 |
| OCRL |
| ODF4 |
| OTUD1 |
| OTUD4 |
| OTUD7B |
| P2RY1 |
| PABPC4L |
| PAFAH1B2 |
| PAICS |
| PALM2-AKAP2 |
| PAPD4 |
| PARK2 |
| PATL1 |
| PCDH10 |
| PDE4A |
| PDRG1 |
| PFN1 |
| PFN2 |
| PGK1 |
| PGM2L1 |
| PGPEP1 |
| PHF13 |
| PHLDA3 |
| PHLPP1 |
| PIGS |
| PIK3R3 |
| PKP1 |
| PLAU |
| PLEKHF2 |
| PLXNC1 |
| PNRC1 |
| POGZ |
| POLI |
| PPARA |
| PPP1R15B |
| PPP2R5E |
| PPP6R1 |
| PPTC7 |
| PRICKLE2 |
| PRKAA1 |
| PRKACB |
| PRMT5 |
| PRR14L |
| PRRC2C |
| PRRG4 |
| PRUNE2 |
| PSAP |
| PSMG2 |
| PTBP2 |
| PTCD2 |
| PTEN |
| PTPRB |
| PTPRG |
| PURG |
| QKI |
| RAB13 |
| RAB14 |
| RAB18 |
| RAB1A |
| RAB21 |
| RAB2B |
| RAB34 |
| RAB5B |
| RAB8B |
| RACGAP1 |
| RAF1 |
| RAN |
| RAP1A |
| RAP1B |
| RAP2C |
| RAPGEF2 |
| RAPGEF4 |
| RAPGEF6 |
| RASA1 |
| RASSF2 |
| RASSF5 |
| RASSF8 |
| RBBP8 |
| RBM20 |
| RBM25 |
| RBM38 |
| RCOR1 |
| REEP3 |
| RGL1 |
| RGL2 |
| RHOB |
| RHOBTB3 |
| RLIM |
| RNF11 |
| RNF111 |
| RNF167 |
| RNF216 |
| RNF4 |
| RNF44 |
| RORA |
| RPF2 |
| RPLP0 |
| RPS4Y1 |
| RPS6KA5 |
| RRAGD |
| RRAS2 |
| S1PR2 |
| SAMD8 |
| SATB1 |
| SBF2 |
| SBNO1 |
| SCD |
| SDE2 |
| SEC61A1 |
| SEC63 |
| SECISBP2L |
| SEL1L |
| SEMA4C |
| SEPHS2 |
| SERBP1 |
| SERINC3 |
| SESN3 |
| SF3A1 |
| SF3B3 |
| SFTPA1 |
| SGK1 |
| SH3KBP1 |
| SHCBP1 |
| SIVA1 |
| SKIL |
| SLC12A7 |
| SLC25A12 |
| SLC27A1 |
| SLC30A7 |
| SLC37A1 |
| SLC38A2 |
| SLC38A7 |
| SLC39A9 |
| SLC46A1 |
| SLC48A1 |
| SLC6A8 |
| SLC7A11 |
| SLC9A1 |
| SLC9A6 |
| SMAD4 |
| SMAD5 |
| SMARCA2 |
| SMG1 |
| SMOC1 |
| SNAP25 |
| SNX17 |
| SNX5 |
| SOCS1 |
| SOCS3 |
| SOCS7 |
| SOX4 |
| SOX6 |
| SPATA2 |
| SPG20 |
| SPTSSA |
| SRPX2 |
| STAT5B |
| STK38 |
| STK4 |
| STOX2 |
| STX12 |
| STX16 |
| STX6 |
| STXBP4 |
| SUZ12 |
| TAF4 |
| TBRG1 |
| TCN2 |
| TF |
| TFB1M |
| TGFBR2 |
| TGIF1 |
| TGOLN2 |
| THBS1 |
| TLR2 |
| TMBIM6 |
| TMEM106B |
| TMEM117 |
| TMEM138 |
| TMEM2 |
| TMEM59 |
| TMEM64 |
| TMEM87A |
| TMEM9B |
| TMOD3 |
| TMTC1 |
| TNF |
| TNFAIP3 |
| TNFAIP6 |
| TNFRSF10B |
| TNFRSF11A |
| TNFRSF12A |
| TNFRSF1B |
| TNIP1 |
| TNKS |
| TNPO2 |
| TNRC6A |
| TNRC6B |
| TP53 |
| TP53INP1 |
| TRAK2 |
| TRIM33 |
| TRIM37 |
| TRPC3 |
| TXLNG |
| UBE2A |
| UBE2D3 |
| UBL3 |
| UBN2 |
| USP13 |
| USP37 |
| USP8 |
| VAMP1 |
| VAMP3 |
| VPS37A |
| VPS37B |
| VPS4B |
| WAC |
| WASL |
| WBP2 |
| WBP4 |
| WDFY3 |
| WDR1 |
| WDR20 |
| WDR26 |
| WDR45B |
| WDR96 |
| WNK1 |
| WNK3 |
| WNT10A |
| WNT7B |
| XIAP |
| YIPF6 |
| YTHDF1 |
| YY1 |
| ZBTB18 |
| ZBTB4 |
| ZBTB47 |
| ZBTB7B |
| ZDHHC18 |
| ZDHHC7 |
| ZFAND1 |
| ZFAND5 |
| ZFP14 |
| ZFYVE26 |
| ZFYVE9 |
| ZIC5 |
| ZMAT3 |
| ZMYND11 |
| ZNF107 |
| ZNF134 |
| ZNF138 |
| ZNF154 |
| ZNF217 |
| ZNF28 |
| ZNF366 |
| ZNF367 |
| ZNF384 |
| ZNF526 |
| ZNF544 |
| ZNF620 |
| ZNF644 |
| ZNF680 |
| ZNF721 |
| ZNF772 |
| ZNF799 |
| ZNF800 |
| ZNF846 |
| ZNF99 |
| ZSCAN2 |
