# Supplementary material for: The Long-Term Effects of Early Life Stress on the Modulation of miR-19 Levels
Source: Front Psychiatry. 2020 May 15;11:389. doi: 10.3389/fpsyt.2020.00389 (PMC7243913; doi:10.3389/fpsyt.2020.00389)
Supplement: Supplementary Table 4 — 229 statistically significant pathways regulated by miR-19a. [file Table_4.docx]

**Supplementary Table 4.**

| Ingenuity Canonical Pathways | -log(p-value) | Ratio | Molecules |
| --- | --- | --- | --- |
| Molecular Mechanisms of Cancer | 7,35 | 0,0885 | AKT1,BCL2L11,BMP3,BMPR2,CASP10,CCND1,CCND2,CHEK1,CHEK2,E2F8,FAS,FOS,FZD6,ITGA2,MAP2K3,MAPK1,MAPK14,  MYC,NF1,PIK3R3,PRKACB,RAF1,RAP1A,RAP1B,RASA1,RHOB,RRAS2,SMAD4,SMAD5,TGFBR2,TP53,WNT10A,WNT7B,XIAP |
| Germ Cell-Sertoli Cell Junction Signaling | 7,27 | 0,126 | ACTB,AKT1,CFL2,EPN2,ITGA2,MAP2K3,MAP3K1,MAP3K14,MAP3K3,MAP3K9,MAPK1,MAPK14,PIK3R3,RAB8B,RAP1A,RAP1B,  RHOB,RRAS2,TGFBR2,TNF,WASL |
| B Cell Receptor Signaling | 6,66 | 0,116 | AKT1,CALM1 (includes others),CD22,CFL2,ETS1,MALT1,MAP2K3,MAP3K1,MAP3K14,MAP3K3,MAP3K9,MAPK1,MAPK14,OCRL,  PIK3R3,PTEN,RAF1,RAP1A,RAP1B,RASSF5,RRAS2 |
| TGF-β Signaling | 6,6 | 0,156 | BMPR2,FOS,MAP2K3,MAPK1,MAPK14,RAF1,RAP1A,RAP1B,RNF111,RRAS2,SMAD4,SMAD5,TGFBR2,TGIF1,ZFYVE9 |
| p53 Signaling | 6,47 | 0,153 | AKT1,CCND1,CCND2,CHEK1,CHEK2,FAS,KAT2B,MAPK14,MDM4,PIK3R3,PTEN,THBS1,TNFRSF10B,TP53,TP53INP1 |
| Chronic Myeloid Leukemia Signaling | 6,19 | 0,146 | AKT1,CCND1,E2F8,HDAC4,MAPK1,MYC,PIK3R3,RAF1,RAP1A,RAP1B,RRAS2,SMAD4,STAT5B,TGFBR2,TP53 |
| Mouse Embryonic Stem Cell Pluripotency | 6,19 | 0,146 | AKT1,BMPR2,FZD6,MAPK1,MAPK14,MYC,PIK3R3,RAF1,RAP1A,RAP1B,RRAS2,SMAD4,SMAD5,TP53,XIAP |
| PPARα/RXRα Activation | 6,15 | 0,112 | ABCA1,ADIPOR2,BCL3,BMPR2,CLOCK,MAP2K3,MAP3K14,MAPK1,MAPK14,PPARA,PRKAA1,PRKACB,RAF1,RAP1A,RAP1B,  RRAS2,SLC27A1,SMAD4,STAT5B,TGFBR2 |
| HGF Signaling | 5,76 | 0,135 | AKT1,CCND1,ETS1,FOS,ITGA2,MAP3K1,MAP3K14,MAP3K3,MAP3K9,MAPK1,PIK3R3,RAF1,RAP1A,RAP1B,RRAS2 |
| IL-6 Signaling | 5,74 | 0,127 | AKT1,FOS,MAP2K3,MAP3K14,MAPK1,MAPK14,PIK3R3,RAF1,RAP1A,RAP1B,RRAS2,SOCS1,SOCS3,TNF,TNFAIP6,TNFRSF1B |
| Endometrial Cancer Signaling | 5,63 | 0,18 | AKT1,CCND1,MAPK1,MYC,PIK3R3,PTEN,RAF1,RAP1A,RAP1B,RRAS2,TP53 |
| NF-κB Signaling | 5,57 | 0,107 | AKT1,BMPR2,MALT1,MAP3K1,MAP3K14,MAP3K3,PIK3R3,PRKACB,RAF1,RAP1A,RAP1B,RRAS2,TGFBR2,TLR2,TNF,TNFAIP3,  TNFRSF11A,TNFRSF1B,TNIP1 |
| Non-Small Cell Lung Cancer Signaling | 5,57 | 0,162 | AKT1,CCND1,ITPR1,MAPK1,PIK3R3,RAF1,RAP1A,RAP1B,RASSF5,RRAS2,STK4,TP53 |
| RANK Signaling in Osteoclasts | 5,57 | 0,149 | AKT1,CALM1 (includes others),FOS,MAP3K1,MAP3K14,MAP3K3,MAP3K9,MAPK1,MAPK14,PIK3R3,RAF1,TNFRSF11A,XIAP |
| Melanoma Signaling | 5,51 | 0,196 | AKT1,CCND1,MAPK1,PIK3R3,PTEN,RAF1,RAP1A,RAP1B,RRAS2,TP53 |
| Glioblastoma Multiforme Signaling | 5,49 | 0,11 | AKT1,CCND1,E2F8,FZD6,ITPR1,MAPK1,MYC,NF1,PIK3R3,PTEN,RAF1,RAP1A,RAP1B,RHOB,RRAS2,TP53,WNT10A,WNT7B |
| Ceramide Signaling | 5,46 | 0,146 | AKT1,CERK,FOS,MAP3K1,PIK3R3,PPP2R5E,RAF1,RAP1A,RAP1B,RRAS2,S1PR2,TNF,TNFRSF1B |
| Acute Myeloid Leukemia Signaling | 5,46 | 0,146 | AKT1,CCND1,KIT,KITLG,MAP2K3,MAPK1,MYC,PIK3R3,RAF1,RAP1A,RAP1B,RRAS2,STAT5B |
| Sertoli Cell-Sertoli Cell Junction Signaling | 5,43 | 0,105 | ACTB,AKT1,EPN2,ITGA2,MAP2K3,MAP3K1,MAP3K14,MAP3K3,MAP3K9,MAPK1,MAPK14,PRKACB,PTEN,RAB8B,RAF1,RAP1A,  RAP1B,RRAS2,TNF |
| Role of NANOG in Mammalian Embryonic Stem Cell Pluripotency | 5,43 | 0,127 | AKT1,BMP3,BMPR2,FZD6,MAPK1,PIK3R3,RAF1,RAP1A,RAP1B,RRAS2,SMAD4,SMAD5,TP53,WNT10A,WNT7B |
| IGF-1 Signaling | 5,35 | 0,133 | AKT1,FOS,GRB10,MAPK1,PIK3R3,PRKACB,RAF1,RAP1A,RAP1B,RASA1,RRAS2,SOCS1,SOCS3,SOCS7 |
| ErbB2-ErbB3 Signaling | 5,34 | 0,169 | AKT1,CCND1,MAPK1,MYC,PIK3R3,PTEN,RAF1,RAP1A,RAP1B,RRAS2,STAT5B |
| Glucocorticoid Receptor Signaling | 5,21 | 0,0808 | ACTB,AKT1,ARID1A,ESR1,FOS,GTF2E2,GTF2H5,KAT2B,MAP3K1,MAP3K14,MAPK1,MAPK14,PIK3R3,PLAU,PRKAA1,PRKACB,  RAF1,RAP1A,RAP1B,RRAS2,SGK1,SMAD4,SMARCA2,STAT5B,TAF4,TGFBR2,TNF |
| JAK/Stat Signaling | 5,15 | 0,148 | AKT1,FOS,MAPK1,PIK3R3,RAF1,RAP1A,RAP1B,RRAS2,SOCS1,SOCS3,SOCS7,STAT5B |
| Prolactin Signaling | 5,09 | 0,146 | FOS,MAPK1,MYC,PIK3R3,RAF1,RAP1A,RAP1B,RRAS2,SOCS1,SOCS3,SOCS7,STAT5B |
| RAR Activation | 5,09 | 0,0995 | ACTB,AKT1,ARID1A,FOS,GTF2H5,KAT2B,MAP3K1,MAPK1,MAPK14,PIK3R3,PNRC1,PRKACB,PTEN,SMAD4,SMAD5,SMARCA2,  STAT5B,TAF4,TNIP1 |
| PTEN Signaling | 5,07 | 0,119 | AKT1,BCL2L11,BMPR2,CCND1,ITGA2,MAPK1,OCRL,PIK3R3,PTEN,RAF1,RAP1A,RAP1B,RRAS2,TGFBR2,TNFRSF11A |
| BMP signaling pathway | 5,04 | 0,145 | BMP3,BMPR2,MAPK1,MAPK14,PRKACB,RAF1,RAP1A,RAP1B,RRAS2,SMAD4,SMAD5,XIAP |
| Apoptosis Signaling | 4,99 | 0,133 | BCL2L11,CASP10,FAS,MAP3K14,MAPK1,RAF1,RAP1A,RAP1B,RRAS2,TNF,TNFRSF1B,TP53,XIAP |
| Acute Phase Response Signaling | 4,95 | 0,101 | AKT1,FOS,MAP2K3,MAP3K1,MAP3K14,MAPK1,MAPK14,PIK3R3,RAF1,RAP1A,RAP1B,RRAS2,SOCS1,SOCS3,SOCS7,TF,TNF,  TNFRSF1B |
| ERK5 Signaling | 4,9 | 0,153 | AKT1,FOS,MAP3K3,MEF2A,MYC,RAP1A,RAP1B,RPS6KA5,RRAS2,SGK1,WNK1 |
| Role of Osteoblasts, Osteoclasts and Chondrocytes in Rheumatoid Arthritis | 4,86 | 0,0926 | AKT1,BMP3,BMPR2,CALM1 (includes others),FOS,FZD6,ITGA2,MAP2K3,MAP3K14,MAPK1,MAPK14,PIK3R3,SMAD4,SMAD5,TNF,  TNFRSF11A,TNFRSF1B,WNT10A,WNT7B,XIAP |
| IL-2 Signaling | 4,72 | 0,161 | AKT1,FOS,MAPK1,PIK3R3,RAF1,RAP1A,RAP1B,RRAS2,SOCS1,STAT5B |
| TNFR2 Signaling | 4,64 | 0,241 | FOS,MAP3K1,MAP3K14,TNF,TNFAIP3,TNFRSF1B,XIAP |
| Aryl Hydrocarbon Receptor Signaling | 4,63 | 0,109 | CCNA2,CCND1,CCND2,CHEK1,CHEK2,ESR1,ESR2,FAS,FOS,MAPK1,MYC,NFIA,NFIB,TNF,TP53 |
| Hereditary Breast Cancer Signaling | 4,63 | 0,109 | ACTB,AKT1,ARID1A,CCND1,CHEK1,CHEK2,FANCF,HDAC4,PIK3R3,PTEN,RAP1A,RAP1B,RRAS2,SMARCA2,TP53 |
| Erythropoietin Signaling | 4,62 | 0,143 | AKT1,FOS,MAPK1,PIK3R3,RAF1,RAP1A,RAP1B,RRAS2,SOCS1,SOCS3,STAT5B |
| GDNF Family Ligand-Receptor Interactions | 4,62 | 0,143 | FOS,FRS2,GDNF,ITPR1,MAPK1,PIK3R3,RAF1,RAP1A,RAP1B,RASA1,RRAS2 |
| Ovarian Cancer Signaling | 4,56 | 0,108 | AKT1,CCND1,EDN1,FZD6,MAPK1,PIK3R3,PRKACB,PTEN,RAF1,RAP1A,RAP1B,RRAS2,TP53,WNT10A,WNT7B |
| Colorectal Cancer Metastasis Signaling | 4,51 | 0,085 | AKT1,CCND1,DCC,FOS,FZD6,GRK3,MAPK1,MYC,PIK3R3,PRKACB,RAP1A,RAP1B,RHOB,RRAS2,SMAD4,TGFBR2,TLR2,TNF,  TP53,WNT10A,WNT7B |
| Type II Diabetes Mellitus Signaling | 4,48 | 0,106 | ACSL4,ADIPOR2,AKT1,CACNB2,MAP3K1,MAP3K14,MAPK1,PIK3R3,PRKAA1,SLC27A1,SOCS1,SOCS3,SOCS7,TNF,TNFRSF1B |
| Neuregulin Signaling | 4,48 | 0,128 | AKT1,ERBB4,ITGA2,MAPK1,MYC,PIK3R3,PTEN,RAF1,RAP1A,RAP1B,RRAS2,STAT5B |
| Cardiac Hypertrophy Signaling (Enhanced) | 4,42 | 0,0672 | AKT1,BMPR2,CALM1 (includes others),EDN1,FZD6,GDPD1,HDAC4,ITGA2,ITPR1,MAP2K3,MAP3K1,MAP3K14,MAP3K3,MAP3K9,MAPK1,MAPK14,  MEF2A,MYC,PDE4A,PIK3R3,PRKACB,PTEN,RAF1,RAP1A,RAP1B,RPS6KA5,RRAS2,TGFBR2,TNF,TNFRSF1B,WNT10A,WNT7B |
| PEDF Signaling | 4,31 | 0,133 | AKT1,FAS,GDNF,MAPK1,MAPK14,PIK3R3,RAF1,RAP1A,RAP1B,RRAS2,TP53 |
| EGF Signaling | 4,29 | 0,161 | AKT1,FOS,ITPR1,MAP3K1,MAPK1,MAPK14,PIK3R3,RAF1,RASA1 |
| NGF Signaling | 4,28 | 0,114 | AKT1,MAP3K1,MAP3K14,MAP3K3,MAP3K9,MAPK1,PIK3R3,RAF1,RAP1A,RAP1B,RPS6KA5,RRAS2,TP53 |
| GM-CSF Signaling | 4,19 | 0,141 | AKT1,CCND1,ETS1,MAPK1,PIK3R3,RAF1,RAP1A,RAP1B,RRAS2,STAT5B |
| Sumoylation Pathway | 4,17 | 0,119 | ETS1,FAS,FOS,RAN,RCOR1,RHOB,RNF4,SERBP1,SMAD4,TP53,XIAP,ZNF217 |
| Cancer Drug Resistance By Drug Efflux | 4,17 | 0,155 | AKT1,MAPK1,PIK3R3,PTEN,RAF1,RAP1A,RAP1B,RRAS2,TP53 |
| Production of Nitric Oxide and Reactive Oxygen Species in Macrophages | 4,15 | 0,0914 | AKT1,FOS,MAP3K1,MAP3K14,MAP3K3,MAP3K9,MAPK1,MAPK14,PIK3R3,PPARA,PPP2R5E,RAP1A,RAP1B,RHOB,TLR2,TNF,  TNFRSF1B |
| IL-15 Signaling | 4,14 | 0,139 | AKT1,MAPK1,MAPK14,PIK3R3,RAF1,RAP1A,RAP1B,RRAS2,STAT5B,TNF |
| PDGF Signaling | 4,11 | 0,126 | FOS,MAP3K1,MAPK1,MYC,OCRL,PIK3R3,RAF1,RAP1A,RAP1B,RASA1,RRAS2 |
| STAT3 Pathway | 4,1 | 0,104 | BMPR2,MAP3K9,MAPK1,MAPK14,MYC,RAF1,RAP1A,RAP1B,RRAS2,SOCS1,SOCS3,SOCS7,TGFBR2,TNFRSF11A |
| GNRH Signaling | 4,09 | 0,0941 | CACNB2,CALM1 (includes others),FOS,ITPR1,MAP2K3,MAP3K1,MAP3K14,MAP3K3,MAP3K9,MAPK1,MAPK14,PRKACB,RAF1,  RAP1A,RAP1B,RRAS2 |
| Regulation of IL-2 Expression in Activated and Anergic T Lymphocytes | 4,07 | 0,125 | CALM1 (includes others),FOS,MALT1,MAP3K1,MAPK1,RAF1,RAP1A,RAP1B,RRAS2,SMAD4,TGFBR2 |
| Insulin Receptor Signaling | 4,07 | 0,103 | AKT1,GRB10,MAPK1,OCRL,PIK3R3,PRKACB,PTEN,RAF1,RAP1A,RAP1B,RRAS2,SGK1,SOCS3,STXBP4 |
| Estrogen-Dependent Breast Cancer Signaling | 4,04 | 0,135 | AKT1,CCND1,ESR1,FOS,MAPK1,PIK3R3,RAP1A,RAP1B,RRAS2,STAT5B |
| Telomerase Signaling | 4 | 0,114 | AKT1,ETS1,HDAC4,MAPK1,MYC,PIK3R3,PPP2R5E,RAF1,RAP1A,RAP1B,RRAS2,TP53 |
| Toll-like Receptor Signaling | 3,99 | 0,133 | FOS,MAP2K3,MAP3K1,MAP3K14,MAPK1,MAPK14,PPARA,TLR2,TNF,TNFAIP3 |
| Estrogen-mediated S-phase Entry | 3,93 | 0,231 | CCNA2,CCND1,E2F8,ESR1,ESR2,MYC |
| Neurotrophin/TRK Signaling | 3,89 | 0,13 | AKT1,FOS,FRS2,MAP2K3,MAPK1,PIK3R3,RAF1,RAP1A,RAP1B,RRAS2 |
| Thyroid Cancer Signaling | 3,86 | 0,16 | CCND1,GDNF,MAPK1,MYC,RAP1A,RAP1B,RRAS2,TP53 |
| Thrombopoietin Signaling | 3,82 | 0,141 | FOS,MAPK1,MYC,PIK3R3,RAF1,RAP1A,RAP1B,RRAS2,STAT5B |
| Cardiac Hypertrophy Signaling | 3,82 | 0,0805 | ADSS,AKT1,CALM1 (includes others),MAP2K3,MAP3K1,MAP3K14,MAP3K3,MAP3K9,MAPK1,MAPK14,MEF2A,PIK3R3,  PRKACB,RAF1,RAP1A,RAP1B,RHOB,RRAS2,TGFBR2 |
| Glioma Signaling | 3,81 | 0,109 | AKT1,CALM1 (includes others),CCND1,E2F8,MAPK1,PIK3R3,PTEN,RAF1,RAP1A,RAP1B,RRAS2,TP53 |
| ErbB Signaling | 3,81 | 0,117 | AKT1,ERBB4,FOS,MAP2K3,MAPK1,MAPK14,PIK3R3,RAF1,RAP1A,RAP1B,RRAS2 |
| UVC-Induced MAPK Signaling | 3,8 | 0,157 | FOS,MAPK1,MAPK14,RAF1,RAP1A,RAP1B,RRAS2,TP53 |
| FAK Signaling | 3,77 | 0,116 | ACTB,AKT1,GIT2,ITGA2,MAPK1,PIK3R3,PTEN,RAF1,RAP1A,RAP1B,RRAS2 |
| FLT3 Signaling in Hematopoietic Progenitor Cells | 3,75 | 0,125 | AKT1,MAPK1,MAPK14,PIK3R3,RAF1,RAP1A,RAP1B,RPS6KA5,RRAS2,STAT5B |
| Role of Macrophages, Fibroblasts and Endothelial Cells in Rheumatoid Arthritis | 3,72 | 0,0731 | AKT1,CALM1 (includes others),CCND1,FOS,FZD6,MAP2K3,MAP3K14,MAPK1,MAPK14,MYC,PIK3R3,RAF1,RAP1A,RAP1B,  RRAS2,SOCS1,SOCS3,TLR2,TNF,TNFRSF1B,WNT10A,WNT7B |
| PI3K/AKT Signaling | 3,69 | 0,1 | AKT1,CCND1,ITGA2,MAPK1,OCRL,PIK3R3,PPP2R5E,PTEN,RAF1,RAP1A,RAP1B,RRAS2,TP53 |
| Apelin Endothelial Signaling Pathway | 3,62 | 0,104 | AKT1,CALM1 (includes others),FOS,HDAC4,MAPK1,MEF2A,PIK3R3,PRKAA1,RAF1,RAP1A,RAP1B,RRAS2 |
| LPS-stimulated MAPK Signaling | 3,62 | 0,12 | FOS,MAP2K3,MAP3K14,MAPK1,MAPK14,PIK3R3,RAF1,RAP1A,RAP1B,RRAS2 |
| NF-κB Activation by Viruses | 3,62 | 0,12 | AKT1,ITGA2,MAP3K1,MAP3K14,MAPK1,PIK3R3,RAF1,RAP1A,RAP1B,RRAS2 |
| Endocannabinoid Developing Neuron Pathway | 3,59 | 0,103 | AKT1,CCND1,MAP2K3,MAPK1,MAPK14,MAPK6,PIK3R3,PRKACB,RAF1,RAP1A,RAP1B,RRAS2 |
| Cholecystokinin/Gastrin-mediated Signaling | 3,55 | 0,103 | FOS,ITPR1,MAP2K3,MAPK1,MAPK14,MEF2A,RAF1,RAP1A,RAP1B,RHOB,RRAS2,TNF |
| Role of Tissue Factor in Cancer | 3,55 | 0,103 | AKT1,CFL2,MAPK1,MAPK14,PIK3R3,PTEN,RAP1A,RAP1B,RPS6KA5,RRAS2,STAT5B,TP53 |
| FGF Signaling | 3,53 | 0,118 | AKT1,FRS2,ITPR1,MAP2K3,MAP3K1,MAPK1,MAPK14,PIK3R3,RAF1,RPS6KA5 |
| PPAR Signaling | 3,53 | 0,109 | FOS,MAP3K14,MAPK1,PPARA,RAF1,RAP1A,RAP1B,RRAS2,STAT5B,TNF,TNFRSF1B |
| Renin-Angiotensin Signaling | 3,52 | 0,102 | FOS,ITPR1,MAP3K1,MAPK1,MAPK14,PIK3R3,PRKACB,RAF1,RAP1A,RAP1B,RRAS2,TNF |
| Oncostatin M Signaling | 3,49 | 0,163 | MAPK1,PLAU,RAF1,RAP1A,RAP1B,RRAS2,STAT5B |
| Regulation of the Epithelial-Mesenchymal Transition Pathway | 3,48 | 0,0833 | AKT1,ETS1,FRS2,FZD6,HMGA2,MAP2K3,MAPK1,PIK3R3,RAF1,RAP1A,RAP1B,RRAS2,SMAD4,TGFBR2,WNT10A,  WNT7B |
| Regulation of eIF4 and p70S6K Signaling | 3,47 | 0,0903 | AGO1,AGO3,AKT1,EIF4A2,ITGA2,MAPK1,MAPK14,PIK3R3,PPP2R5E,RAF1,RAP1A,RAP1B,RPS4Y1,RRAS2 |
| Estrogen Receptor Signaling | 3,47 | 0,0949 | ESR1,ESR2,G6PC,GTF2H5,KAT2B,MAPK1,MED12L,MED21,RAF1,RAP1A,RAP1B,RRAS2,TAF4 |
| AMPK Signaling | 3,46 | 0,0802 | ACTB,AKT1,ARID1A,CAB39,CCNA2,CCND1,KAT2B,MAP2K3,MAPK1,MAPK14,PHLPP1,PIK3R3,PPP2R5E,PRKAA1,  PRKACB,RAB1A,SMARCA2 |
| Role of NFAT in Cardiac Hypertrophy | 3,46 | 0,0802 | AKT1,CACNB2,CALM1 (includes others),HDAC4,ITPR1,MAP2K3,MAP3K1,MAPK1,MAPK14,MEF2A,PIK3R3,PRKACB,  RAF1,RAP1A,RAP1B,  RRAS2,TGFBR2 |
| CNTF Signaling | 3,45 | 0,14 | AKT1,MAPK1,PIK3R3,RAF1,RAP1A,RAP1B,RPS6KA5,RRAS2 |
| ERK/MAPK Signaling | 3,45 | 0,0829 | ESR1,ETS1,FOS,ITGA2,MAPK1,MYC,MYCN,PIK3R3,PPP2R5E,PRKACB,RAF1,RAP1A,RAP1B,RAPGEF4,RPS6KA5,RRAS2 |
| EIF2 Signaling | 3,42 | 0,0794 | ACTB,AGO1,AGO3,AKT1,CCND1,EIF4A2,MAPK1,MYC,MYCN,PIK3R3,RAF1,RAP1A,RAP1B,RPLP0,RPS4Y1,RRAS2,XIAP |
| Inhibition of Angiogenesis by TSP1 | 3,4 | 0,188 | AKT1,MAPK1,MAPK14,TGFBR2,THBS1,TP53 |
| T Cell Receptor Signaling | 3,38 | 0,105 | CALM1 (includes others),FOS,MALT1,MAP3K1,MAPK1,PIK3R3,RAF1,RAP1A,RAP1B,RASA1,RRAS2 |
| Prostate Cancer Signaling | 3,33 | 0,111 | AKT1,CCND1,MAPK1,PIK3R3,PTEN,RAF1,RAP1A,RAP1B,RRAS2,TP53 |
| FAT10 Cancer Signaling Pathway | 3,3 | 0,152 | AKT1,BMPR2,SMAD4,TGFBR2,TNF,TNFRSF1B,TP53 |
| Role of MAPK Signaling in the Pathogenesis of Influenza | 3,25 | 0,118 | AKT1,MAP2K3,MAPK1,MAPK14,RAF1,RAP1A,RAP1B,RRAS2,TNF |
| Sirtuin Signaling Pathway | 3,25 | 0,0707 | ABCA1,AKT1,ATG14,ATG16L1,ATG2B,ATG5,BCL2L11,CLOCK,MAPK1,MAPK6,MYC,MYCN,NDRG1,PGK1,PPARA,PRKAA1,  RBBP8,SF3A1,  TNF,TP53 |
| Chemokine Signaling | 3,21 | 0,117 | CALM1 (includes others),FOS,MAPK1,MAPK14,MPRIP,RAF1,RAP1A,RAP1B,RRAS2 |
| Melanocyte Development and Pigmentation Signaling | 3,14 | 0,105 | KIT,KITLG,MAPK1,PIK3R3,PRKACB,RAF1,RAP1A,RAP1B,RPS6KA5,RRAS2 |
| Tight Junction Signaling | 3,14 | 0,0838 | ACTB,AKT1,FOS,NAPB,PPP2R5E,PRKACB,PTEN,RAB13,SNAP25,STX16,TGFBR2,TNF,TNFRSF1B,VAMP3 |
| IL-22 Signaling | 3,13 | 0,208 | AKT1,MAPK1,MAPK14,SOCS3,STAT5B |
| Renal Cell Carcinoma Signaling | 3,13 | 0,114 | AKT1,ETS1,FOS,MAPK1,PIK3R3,RAF1,RAP1A,RAP1B,RRAS2 |
| Pyridoxal 5'-phosphate Salvage Pathway | 3,11 | 0,125 | G6PC,GRK6,MAP2K3,MAP3K9,MAPK1,MAPK6,PRKAA1,SGK1 |
| Bladder Cancer Signaling | 3,11 | 0,104 | CCND1,MAPK1,MYC,RAF1,RAP1A,RAP1B,RPS6KA5,RRAS2,THBS1,TP53 |
| Role of BRCA1 in DNA Damage Response | 3,09 | 0,112 | ACTB,ARID1A,CHEK1,CHEK2,E2F8,FANCF,RBBP8,SMARCA2,TP53 |
| IL-3 Signaling | 3,09 | 0,112 | AKT1,FOS,MAPK1,PIK3R3,RAF1,RAP1A,RAP1B,RRAS2,STAT5B |
| Adipogenesis pathway | 3,08 | 0,0909 | AKT1,ATG5,BMPR2,CLOCK,FZD6,GTF2H5,HDAC4,KAT2B,SMAD5,STAT5B,TNF,TP53 |
| PAK Signaling | 3,07 | 0,103 | CFL2,ITGA2,MAPK1,PIK3R3,RAF1,RAP1A,RAP1B,RRAS2,TNF,WASL |
| UVA-Induced MAPK Signaling | 3,07 | 0,103 | FOS,MAPK1,MAPK14,PIK3R3,RAP1A,RAP1B,RPS6KA5,RRAS2,TNKS,TP53 |
| IL-17 Signaling | 3,05 | 0,111 | AKT1,MAP2K3,MAP3K14,MAPK1,MAPK14,PIK3R3,RAP1A,RAP1B,RRAS2 |
| Role of JAK family kinases in IL-6-type Cytokine Signaling | 3,05 | 0,2 | MAPK1,MAPK14,SOCS1,SOCS3,STAT5B |
| Myc Mediated Apoptosis Signaling | 3,02 | 0,121 | AKT1,FAS,MYC,PIK3R3,RAP1A,RAP1B,RRAS2,TP53 |
| Adrenomedullin signaling pathway | 2,98 | 0,0777 | AKT1,CALM1 (includes others),FOS,ITPR1,MAP2K3,MAPK1,MAPK14,MAPK6,PIK3R3,PRKACB,RAF1,RAP1A,RAP1B,  RRAS2,TNF |
| CD27 Signaling in Lymphocytes | 2,97 | 0,135 | FOS,MAP2K3,MAP3K1,MAP3K14,MAP3K3,MAP3K9,SIVA1 |
| ErbB4 Signaling | 2,97 | 0,119 | AKT1,ERBB4,MAPK1,PIK3R3,RAF1,RAP1A,RAP1B,RRAS2 |
| Fc Epsilon RI Signaling | 2,95 | 0,0932 | AKT1,MAP2K3,MAPK1,MAPK14,OCRL,PIK3R3,RAF1,RAP1A,RAP1B,RRAS2,TNF |
| Role of JAK1 and JAK3 in γc Cytokine Signaling | 2,93 | 0,118 | MAPK1,PIK3R3,RAP1A,RAP1B,RRAS2,SOCS1,SOCS3,STAT5B |
| UVB-Induced MAPK Signaling | 2,92 | 0,132 | AKT1,FOS,MAPK1,MAPK14,PIK3R3,RPS6KA5,TP53 |
| HMGB1 Signaling | 2,88 | 0,0823 | AKT1,FOS,KAT2B,MAP2K3,MAPK1,MAPK14,PIK3R3,RAP1A,RAP1B,RHOB,RRAS2,TNF,TNFRSF1B |
| CCR3 Signaling in Eosinophils | 2,87 | 0,0909 | CALM1 (includes others),CFL2,ITPR1,MAPK1,MAPK14,MPRIP,PIK3R3,RAF1,RAP1A,RAP1B,RRAS2 |
| Opioid Signaling Pathway | 2,78 | 0,0697 | AKT1,CACNB2,CALM1 (includes others),FOS,GRK3,GRK6,ITPR1,MAP2K3,MAPK1,MAPK6,MYC,PRKACB,  RAF1,RAP1A,RAP1B,RPS6KA5,RRAS2 |
| Endocannabinoid Cancer Inhibition Pathway | 2,77 | 0,0839 | AKT1,CASP10,CCND1,CCND2,MAP2K3,MAPK1,MAPK14,MYC,PIK3R3,PRKAA1,PRKACB,RAF1 |
| Growth Hormone Signaling | 2,77 | 0,111 | FOS,MAPK1,PIK3R3,RPS6KA5,SOCS1,SOCS3,SOCS7,STAT5B |
| CDK5 Signaling | 2,74 | 0,0935 | ITGA2,MAPK1,MAPK14,MAPK6,PPP2R5E,PRKACB,RAF1,RAP1A,RAP1B,RRAS2 |
| Type I Diabetes Mellitus Signaling | 2,74 | 0,0935 | FAS,MAP2K3,MAP3K14,MAPK1,MAPK14,SOCS1,SOCS3,SOCS7,TNF,TNFRSF1B |
| Death Receptor Signaling | 2,72 | 0,1 | ACTB,CASP10,FAS,MAP3K14,TNF,TNFRSF10B,TNFRSF1B,TNKS,XIAP |
| Integrin Signaling | 2,7 | 0,0728 | ACTB,AKT1,ITGA2,MAPK1,MPRIP,PFN1,PFN2,PIK3R3,PTEN,RAF1,RAP1A,RAP1B,RHOB,RRAS2,WASL |
| P2Y Purigenic Receptor Signaling Pathway | 2,7 | 0,0866 | AKT1,FOS,MAPK1,MYC,P2RY1,PIK3R3,PRKACB,RAF1,RAP1A,RAP1B,RRAS2 |
| ILK Signaling | 2,69 | 0,0753 | ACTB,AKT1,CCND1,CFL2,FBLIM1,FOS,MAPK1,MYC,PIK3R3,PPP2R5E,PTEN,RHOB,RPS6KA5,TNF |
| IL-7 Signaling Pathway | 2,69 | 0,108 | AKT1,CCND1,MAPK1,MAPK14,MYC,PIK3R3,SOCS1,STAT5B |
| Angiopoietin Signaling | 2,66 | 0,107 | AKT1,PIK3R3,RAP1A,RAP1B,RASA1,RRAS2,STAT5B,TNIP1 |
| Cdc42 Signaling | 2,64 | 0,0853 | CFL2,CLIP1,EXOC7,FOS,ITGA2,MAPK1,MAPK14,MPRIP,RAF1,RASA1,WASL |
| HIF1α Signaling | 2,6 | 0,0893 | AKT1,EDN1,MAPK1,MAPK14,MAPK6,PIK3R3,RAP1A,RAP1B,RRAS2,TP53 |
| Iron homeostasis signaling pathway | 2,59 | 0,084 | ATP6V0E1,ATP6V1B2,BMP3,BMPR2,MAPK1,SLC46A1,SLC48A1,SMAD4,SMAD5,STAT5B,TF |
| PKCθ Signaling in T Lymphocytes | 2,58 | 0,0795 | CACNB2,FOS,MALT1,MAP3K1,MAP3K14,MAP3K3,MAP3K9,MAPK1,PIK3R3,RAP1A,RAP1B,RRAS2 |
| Human Embryonic Stem Cell Pluripotency | 2,54 | 0,0827 | AKT1,BMP3,BMPR2,FZD6,PIK3R3,S1PR2,SMAD4,SMAD5,TGFBR2,WNT10A,WNT7B |
| ATM Signaling | 2,53 | 0,0938 | CBX1,CBX5,CHEK1,CHEK2,MAPK14,MDM4,PPP2R5E,RBBP8,TP53 |
| CDP-diacylglycerol Biosynthesis I | 2,53 | 0,2 | ABHD5,AGPAT5,GPAM,LCLAT1 |
| SPINK1 General Cancer Pathway | 2,53 | 0,113 | AKT1,MAPK1,PIK3R3,RAF1,RAP1A,RAP1B,RRAS2 |
| PI3K Signaling in B Lymphocytes | 2,51 | 0,0821 | AKT1,CALM1 (includes others),FOS,ITPR1,MALT1,MAPK1,PTEN,RAF1,RAP1A,RAP1B,RRAS2 |
| Systemic Lupus Erythematosus In T Cell Signaling Pathway | 2,49 | 0,0691 | AKT1,CASP10,DNMT1,ESR1,FAS,FOS,ITPR1,MAP2K3,MAPK1,PIK3R3,PPP2R5E,RAP1A,RAP1B,RHOB,RRAS2 |
| Cyclins and Cell Cycle Regulation | 2,48 | 0,1 | CCNA2,CCND1,CCND2,E2F8,HDAC4,PPP2R5E,RAF1,TP53 |
| TNFR1 Signaling | 2,45 | 0,125 | FOS,MAP3K1,MAP3K14,TNF,TNFAIP3,XIAP |
| Regulation of Cellular Mechanics by Calpain Protease | 2,45 | 0,109 | CCNA2,CCND1,ITGA2,MAPK1,RAP1A,RAP1B,RRAS2 |
| Synaptogenesis Signaling Pathway | 2,44 | 0,0617 | AKT1,CALM1 (includes others),ITPR1,MAPK1,MAPK14,NAPB,PIK3R3,PRKACB,RAB5B,RAF1,RAP1A,RAP1B,  RRAS2,SNAP25,STX16,STXBP4,THBS1,VAMP3,WASL |
| p38 MAPK Signaling | 2,43 | 0,0847 | FAS,MAP2K3,MAPK14,MEF2A,MYC,RPS6KA5,TGFBR2,TNF,TNFRSF1B,TP53 |
| IL-9 Signaling | 2,43 | 0,147 | BCL3,PIK3R3,SOCS3,STAT5B,TNF |
| DNA Methylation and Transcriptional Repression Signaling | 2,43 | 0,147 | DNMT1,HIST2H4A,HIST2H4B,MBD3,MECP2 |
| CD40 Signaling | 2,41 | 0,108 | FOS,MAP2K3,MAP3K14,MAPK1,MAPK14,PIK3R3,TNFAIP3 |
| Purine Nucleotides De Novo Biosynthesis II | 2,39 | 0,273 | ADSS,IMPDH1,PAICS |
| Cell Cycle: G1/S Checkpoint Regulation | 2,37 | 0,106 | CCND1,CCND2,E2F8,HDAC4,MYC,SMAD4,TP53 |
| Phosphatidylglycerol Biosynthesis II (Non-plastidic) | 2,37 | 0,182 | ABHD5,AGPAT5,GPAM,LCLAT1 |
| RhoA Signaling | 2,35 | 0,0826 | ACTB,ARHGAP1,ARHGAP12,CFL2,CIT,MPRIP,PFN1,PFN2,RAPGEF2,RAPGEF6 |
| Corticotropin Releasing Hormone Signaling | 2,35 | 0,078 | CACNB2,CALM1 (includes others),FOS,ITPR1,MAPK1,MAPK14,MEF2A,PRKACB,RAF1,RAP1A,RAP1B |
| Endothelin-1 Signaling | 2,34 | 0,0714 | CASP10,EDN1,FOS,ITPR1,MAPK1,MAPK14,MAPK6,MYC,PIK3R3,RAF1,RAP1A,RAP1B,RRAS2 |
| VEGF Family Ligand-Receptor Interactions | 2,32 | 0,0941 | AKT1,FOS,MAPK1,PIK3R3,RAF1,RAP1A,RAP1B,RRAS2 |
| Osteoarthritis Pathway | 2,27 | 0,0676 | BMPR2,CASP10,FZD6,HDAC4,ITGA2,OCRL,PRKAA1,S1PR2,SMAD4,SMAD5,TGFBR2,TLR2,TNF,TNFRSF1B |
| Cell Cycle Regulation by BTG Family Proteins | 2,27 | 0,135 | BTG1,CCND1,CNOT7,E2F8,PPP2R5E |
| IL-10 Signaling | 2,26 | 0,101 | FOS,MAP2K3,MAP3K14,MAPK1,MAPK14,SOCS3,TNF |
| Paxillin Signaling | 2,22 | 0,0841 | ACTB,GIT2,ITGA2,MAPK1,MAPK14,PIK3R3,RAP1A,RAP1B,RRAS2 |
| Huntington's Disease Signaling | 2,17 | 0,0638 | AKT1,CASP10,HDAC4,ITPR1,MAPK1,NAPB,PIK3R3,RASA1,RCOR1,SGK1,SNAP25,STX16,TAF4,TP53,VAMP3 |
| April Mediated Signaling | 2,17 | 0,128 | FOS,MAP3K1,MAP3K14,MAPK1,MAPK14 |
| Pancreatic Adenocarcinoma Signaling | 2,17 | 0,0826 | AKT1,CCND1,E2F8,MAPK1,PIK3R3,RAF1,SMAD4,TGFBR2,TP53 |
| Wnt/β-catenin Signaling | 2,17 | 0,0706 | AKT1,BMPR2,CCND1,FZD6,MYC,PPP2R5E,SOX4,SOX6,TGFBR2,TP53,WNT10A,WNT7B |
| IL-17A Signaling in Gastric Cells | 2,16 | 0,16 | FOS,MAPK1,MAPK14,TNF |
| Actin Cytoskeleton Signaling | 2,15 | 0,0654 | ACTB,CFL2,ITGA2,MAPK1,MPRIP,PFN1,PFN2,PIK3R3,RAF1,RAP1A,RAP1B,RRAS2,SLC9A1,WASL |
| Epithelial Adherens Junction Signaling | 2,15 | 0,0733 | ACTB,AKT1,BMPR2,CLIP1,EPN2,PTEN,RAP1A,RAP1B,RRAS2,TGFBR2,WASL |
| Glioma Invasiveness Signaling | 2,13 | 0,0959 | MAPK1,PIK3R3,PLAU,RAP1A,RAP1B,RHOB,RRAS2 |
| Rac Signaling | 2,12 | 0,0811 | CFL2,ITGA2,MAP3K1,MAPK1,PIK3R3,RAF1,RAP1A,RAP1B,RRAS2 |
| Gα12/13 Signaling | 2,11 | 0,0763 | AKT1,MAP3K1,MAPK1,MEF2A,PIK3R3,RAF1,RAP1A,RAP1B,RASA1,RRAS2 |
| Antiproliferative Role of TOB in T Cell Signaling | 2,1 | 0,154 | CCNA2,MAPK1,SMAD4,TGFBR2 |
| Role of CHK Proteins in Cell Cycle Checkpoint Control | 2,09 | 0,105 | ATMIN,CHEK1,CHEK2,E2F8,PPP2R5E,TP53 |
| α-Adrenergic Signaling | 2,08 | 0,086 | CALM1 (includes others),ITPR1,MAPK1,PRKACB,RAF1,RAP1A,RAP1B,RRAS2 |
| Role of PKR in Interferon Induction and Antiviral Response | 2,08 | 0,122 | AKT1,MAP2K3,MAPK14,TNF,TP53 |
| B Cell Activating Factor Signaling | 2,08 | 0,122 | FOS,MAP3K1,MAP3K14,MAPK1,MAPK14 |
| Agrin Interactions at Neuromuscular Junction | 2,04 | 0,0921 | ACTB,ERBB4,ITGA2,MAPK1,RAP1A,RAP1B,RRAS2 |
| BAG2 Signaling Pathway | 2,03 | 0,119 | MAPK1,MAPK14,MYC,PRKN,TP53 |
| Salvage Pathways of Pyrimidine Ribonucleotides | 2,03 | 0,0842 | G6PC,GRK6,MAP2K3,MAP3K9,MAPK1,MAPK6,PRKAA1,SGK1 |
| Xenobiotic Metabolism Signaling | 1,98 | 0,0593 | ESD,HDAC4,MAP2K3,MAP3K1,MAP3K14,MAP3K3,MAP3K9,MAPK1,MAPK14,PIK3R3,PPP2R5E,RAF1,  RAP1A,RAP1B,RRAS2,TNF |
| Induction of Apoptosis by HIV1 | 1,98 | 0,1 | FAS,MAP3K14,TNF,TNFRSF1B,TP53,XIAP |
| VEGF Signaling | 1,95 | 0,0816 | ACTB,AKT1,MAPK1,PIK3R3,RAF1,RAP1A,RAP1B,RRAS2 |
| Apelin Cardiomyocyte Signaling Pathway | 1,93 | 0,0808 | AKT1,ITPR1,MAPK1,MAPK14,MAPK6,PIK3R3,SLC9A1,SLC9A6 |
| Phagosome Maturation | 1,92 | 0,0714 | ATP6V0E1,ATP6V1B2,DYNC1LI2,NAPB,RAB5B,SNAP25,STX16,VAMP3,VPS37A,VPS37B |
| Parkinson's Signaling | 1,91 | 0,188 | MAPK1,MAPK14,PRKN |
| IL-23 Signaling Pathway | 1,91 | 0,111 | AKT1,PIK3R3,RORA,SOCS3,TNF |
| PXR/RXR Activation | 1,88 | 0,0952 | AKT1,G6PC,PPARA,PRKACB,SCD,TNF |
| PFKFB4 Signaling Pathway | 1,87 | 0,109 | MAP2K3,MAPK1,PRKACB,RPS6KA5,TP53 |
| NRF2-mediated Oxidative Stress Response | 1,86 | 0,0642 | ACTB,AKT1,FOS,MAP2K3,MAP3K1,MAPK1,MAPK14,PIK3R3,RAF1,RAP1A,RAP1B,RRAS2 |
| SAPK/JNK Signaling | 1,85 | 0,0784 | MAP3K1,MAP3K3,MAP3K9,PIK3R3,RAP1A,RAP1B,RRAS2,TP53 |
| IL-4 Signaling | 1,84 | 0,0843 | AKT1,OCRL,PIK3R3,RAP1A,RAP1B,RRAS2,SOCS1 |
| HER-2 Signaling in Breast Cancer | 1,79 | 0,0824 | AKT1,CCND1,PIK3R3,RAP1A,RAP1B,RRAS2,TP53 |
| Role of JAK2 in Hormone-like Cytokine Signaling | 1,78 | 0,125 | SOCS1,SOCS3,SOCS7,STAT5B |
| T Cell Exhaustion Signaling Pathway | 1,77 | 0,0647 | AKT1,BMPR2,FOS,FOXP1,MAPK1,PIK3R3,PPP2R5E,RAP1A,RAP1B,RRAS2,TGFBR2 |
| 14-3-3-mediated Signaling | 1,76 | 0,0709 | AKT1,FOS,MAPK1,PIK3R3,RAF1,RAP1A,RAP1B,RRAS2,TNF |
| Cell Cycle: G2/M DNA Damage Checkpoint Regulation | 1,76 | 0,102 | CHEK1,CHEK2,KAT2B,MDM4,TP53 |
| Leukocyte Extravasation Signaling | 1,74 | 0,0619 | ACTB,ARHGAP1,ARHGAP12,ITGA2,MAPK1,MAPK14,PIK3R3,RAP1A,RAP1B,RAPGEF4,RASSF5,WASL |
| Regulation of Actin-based Motility by Rho | 1,71 | 0,0795 | ACTB,ITGA2,MPRIP,PFN1,PFN2,RHOB,WASL |
| GADD45 Signaling | 1,7 | 0,158 | CCND1,CCND2,TP53 |
| Axonal Guidance Signaling | 1,69 | 0,0491 | AKT1,BMP3,CFL2,DCC,DPYSL2,FZD6,ITGA2,MAPK1,PFN1,PFN2,PIK3R3,PLXNC1,PRKACB,RAF1,RAP1A,  RAP1B,RASA1,RASSF5,RRAS2,SEMA4C,WASL,WNT10A,WNT7B |
| Melatonin Signaling | 1,67 | 0,0857 | CALM1 (includes others),MAP2K3,MAPK1,PRKACB,RAF1,RORA |
| Actin Nucleation by ARP-WASP Complex | 1,67 | 0,0857 | ITGA2,RAP1A,RAP1B,RHOB,RRAS2,WASL |
| Factors Promoting Cardiogenesis in Vertebrates | 1,66 | 0,0778 | BMP3,BMPR2,FZD6,MAPK14,SMAD4,SMAD5,TGFBR2 |
| Role of NFAT in Regulation of the Immune Response | 1,65 | 0,0621 | AKT1,CALM1 (includes others),FOS,ITPR1,MAPK1,MEF2A,PIK3R3,RAF1,RAP1A,RAP1B,RRAS2 |
| Breast Cancer Regulation by Stathmin1 | 1,65 | 0,06 | CALM1 (includes others),E2F8,ITPR1,MAPK1,PIK3R3,PPP2R5E,PRKACB,RAF1,RAP1A,RAP1B,RRAS2,TP53 |
| IL-1 Signaling | 1,64 | 0,0769 | FOS,MAP2K3,MAP3K1,MAP3K14,MAPK1,MAPK14,PRKACB |
| Ephrin Receptor Signaling | 1,63 | 0,0618 | AKT1,CFL2,ITGA2,MAP3K14,MAPK1,RAF1,RAP1A,RAP1B,RASA1,RRAS2,WASL |
| Small Cell Lung Cancer Signaling | 1,62 | 0,0833 | AKT1,CCND1,MYC,PIK3R3,PTEN,TP53 |
| Hypoxia Signaling in the Cardiovascular System | 1,62 | 0,0833 | AKT1,EDN1,PTEN,TP53,UBE2A,UBE2D3 |
| mTOR Signaling | 1,59 | 0,0588 | AKT1,EIF4A2,MAPK1,PIK3R3,PPP2R5E,PRKAA1,RAP1A,RAP1B,RHOB,RPS4Y1,RPS6KA5,RRAS2 |
| fMLP Signaling in Neutrophils | 1,55 | 0,069 | CALM1 (includes others),ITPR1,MAPK1,PIK3R3,RAF1,RAP1A,RAP1B,RRAS2 |
| Nitric Oxide Signaling in the Cardiovascular System | 1,55 | 0,0737 | AKT1,CALM1 (includes others),ITPR1,MAPK1,PIK3R3,PRKAA1,PRKACB |
| Triacylglycerol Biosynthesis | 1,53 | 0,105 | ABHD5,AGPAT5,GPAM,LCLAT1 |
| Polyamine Regulation in Colon Cancer | 1,53 | 0,136 | AZIN1,MXD1,MYC |
| FcγRIIB Signaling in B Lymphocytes | 1,52 | 0,0789 | AKT1,CACNB2,PIK3R3,RAP1A,RAP1B,RRAS2 |
| Natural Killer Cell Signaling | 1,52 | 0,0678 | AKT1,MAPK1,OCRL,PIK3R3,RAF1,RAP1A,RAP1B,RRAS2 |
| Semaphorin Signaling in Neurons | 1,5 | 0,0877 | ARHGAP1,CFL2,DPYSL2,MAPK1,RHOB |
| CXCR4 Signaling | 1,48 | 0,0606 | AKT1,FOS,ITPR1,MAPK1,PIK3R3,RAF1,RAP1A,RAP1B,RHOB,RRAS2 |
| Superpathway of D-myo-inositol (1,4,5)-trisphosphate Metabolism | 1,48 | 0,13 | IMPAD1,OCRL,PTEN |
| G Beta Gamma Signaling | 1,44 | 0,0656 | AKT1,CACNB2,MAPK1,PRKACB,RAF1,RAP1A,RAP1B,RRAS2 |
| PCP pathway | 1,42 | 0,0833 | FZD6,PFN1,PFN2,WNT10A,WNT7B |
| Signaling by Rho Family GTPases | 1,42 | 0,0539 | ACTB,CFL2,CIT,CLIP1,FOS,ITGA2,MAP3K9,MAPK1,PIK3R3,RAF1,RHOB,SLC9A1,WASL |
| Relaxin Signaling | 1,4 | 0,0612 | AKT1,FOS,GDPD1,MAPK1,PDE4A,PIK3R3,PRKACB,RAP1A,RAP1B |
| Gap Junction Signaling | 1,39 | 0,0564 | ACTB,AKT1,DBN1,ITPR1,MAPK1,PIK3R3,PRKACB,RAF1,RAP1A,RAP1B,RRAS2 |
| IL-8 Signaling | 1,37 | 0,0561 | AKT1,CCND1,CCND2,FOS,MAPK1,PIK3R3,RAF1,RAP1A,RAP1B,RHOB,RRAS2 |
| Synaptic Long Term Potentiation | 1,37 | 0,0635 | CALM1 (includes others),ITPR1,MAPK1,PRKACB,RAF1,RAP1A,RAP1B,RRAS2 |
| Apelin Liver Signaling Pathway | 1,34 | 0,115 | EDN1,FAS,TNF |
| HIPPO signaling | 1,34 | 0,0714 | DLG5,FRMD6,PPP2R5E,SMAD4,SMAD5,STK4 |
| iNOS Signaling | 1,33 | 0,0909 | CALM1 (includes others),FOS,MAPK1,MAPK14 |
| p70S6K Signaling | 1,32 | 0,062 | AKT1,MAPK1,PIK3R3,PPP2R5E,RAF1,RAP1A,RAP1B,RRAS2 |
| eNOS Signaling | 1,31 | 0,0588 | AKT1,CALM1 (includes others),CCNA2,ESR1,ESR2,ITPR1,PIK3R3,PRKAA1,PRKACB |
| Role of Oct4 in Mammalian Embryonic Stem Cell Pluripotency | 1,3 | 0,0889 | IGF2BP1,JARID2,MEF2A,TP53 |
