# Supplementary material for: The Long-Term Effects of Early Life Stress on the Modulation of miR-19 Levels
Source: Front Psychiatry. 2020 May 15;11:389. doi: 10.3389/fpsyt.2020.00389 (PMC7243913; doi:10.3389/fpsyt.2020.00389)
Supplement: Supplementary Table 6 — 210 statistically significant pathways regulated by miR-19b-2. [file Table_6.docx]

**Supplementary Table 6.**

| Ingenuity Canonical Pathways | -log(p-value) | Ratio | Molecules |
| --- | --- | --- | --- |
| TGF-β Signaling | 6,89 | 0,177 | ACVR1,BMPR2,FOS,MAP2K3,MAPK1,MAPK14,RAF1,RAP1A,RAP1B,RNF111,RRAS2,SMAD4,SMAD5,TGFB1,TGFBR2,TGIF1,  ZFYVE9 |
| Prolactin Signaling | 6,34 | 0,183 | ATM,FOS,MAPK1,MYC,NR3C1,PIK3R3,RAF1,RAP1A,RAP1B,RRAS2,SOCS1,SOCS3,SOCS4,SOCS7,STAT5B |
| Molecular Mechanisms of Cancer | 6,09 | 0,0938 | ATM,BCL2L11,BMP3,BMPR2,CASP10,CCND2,CDK19,CHEK1,CHEK2,E2F8,FAS,FOS,FZD6,ITGA2,MAP2K3,MAPK1,MAPK14,  MYC,NF1,PIK3R3,PRKACB,RAF1,RAP1A,RAP1B,RASA1,RHOB,RRAS2,SMAD4,SMAD5,TCF4,TGFB1,TGFBR2,TP53,WNT10A,  WNT7B,XIAP |
| PPARα/RXRα Activation | 5,86 | 0,124 | ACVR1,ADIPOR2,BCL3,BMPR2,CLOCK,MAP2K3,MAP3K14,MAPK1,MAPK14,NCOA3,PPARA,PRKAA1,PRKACB,RAF1,RAP1A,  RAP1B,RRAS2,SLC27A1,SMAD4,STAT5B,TGFB1,TGFBR2 |
| Germ Cell-Sertoli Cell Junction Signaling | 5,74 | 0,126 | ACTB,ATM,CFL2,EPN2,ITGA2,MAP2K3,MAP3K1,MAP3K14,MAP3K3,MAP3K9,MAPK1,MAPK14,PIK3R3,RAB8B,RAP1A,RAP1B,  RHOB,RRAS2,TGFB1,TGFBR2,WASL |
| B Cell Receptor Signaling | 5,74 | 0,122 | ATF2,ATM,CALM1 (includes others),CFL2,CREB1,ETS1,MALT1,MAP2K3,MAP3K1,MAP3K14,MAP3K3,MAP3K9,MAPK1,MAPK14,  OCRL,PIK3R3,PTEN,RAF1,RAP1A,RAP1B,RASSF5,RRAS2 |
| Mouse Embryonic Stem Cell Pluripotency | 5,72 | 0,155 | ATM,BMPR2,FZD6,MAPK1,MAPK14,MYC,PIK3R3,RAF1,RAP1A,RAP1B,RRAS2,SMAD4,SMAD5,TCF4,TP53,XIAP |
| Glucocorticoid Receptor Signaling | 5,68 | 0,0958 | ACTB,ARID1A,ATM,CREB1,ESR1,FOS,GTF2E2,GTF2H5,KAT2B,MAP3K1,MAP3K14,MAPK1,MAPK14,NCOA3,NR3C1,NR3C2,  PIK3R3,PLAU,PRKAA1,PRKACB,RAF1,RAP1A,RAP1B,RRAS2,SGK1,SMAD4,SMARCA2,STAT5B,TAF4,TGFB1,TGFBR2,TSC22D3 |
| BMP signaling pathway | 5,52 | 0,169 | ATF2,BMP3,BMPR2,CREB1,MAPK1,MAPK14,PRKACB,RAF1,RAP1A,RAP1B,RRAS2,SMAD4,SMAD5,XIAP |
| p53 Signaling | 5,32 | 0,153 | ATM,CCND2,CHEK1,CHEK2,FAS,KAT2B,MAPK14,MDM4,PIK3R3,PTEN,ST13,THBS1,TNFRSF10B,TP53,TP53INP1 |
| GDNF Family Ligand-Receptor Interactions | 5,17 | 0,169 | ATM,CREB1,FOS,FRS2,GDNF,ITPR1,MAPK1,PIK3R3,RAF1,RAP1A,RAP1B,RASA1,RRAS2 |
| Chronic Myeloid Leukemia Signaling | 5,05 | 0,146 | ATM,E2F8,HDAC4,MAPK1,MYC,PIK3R3,RAF1,RAP1A,RAP1B,RRAS2,SMAD4,STAT5B,TGFB1,TGFBR2,TP53 |
| IGF-1 Signaling | 4,95 | 0,143 | ATM,FOS,GRB10,MAPK1,PIK3R3,PRKACB,RAF1,RAP1A,RAP1B,RASA1,RRAS2,SOCS1,SOCS3,SOCS4,SOCS7 |
| JAK/Stat Signaling | 4,92 | 0,16 | ATM,FOS,MAPK1,PIK3R3,RAF1,RAP1A,RAP1B,RRAS2,SOCS1,SOCS3,SOCS4,SOCS7,STAT5B |
| LPS-stimulated MAPK Signaling | 4,8 | 0,157 | ATF2,ATM,CREB1,FOS,MAP2K3,MAP3K14,MAPK1,MAPK14,PIK3R3,RAF1,RAP1A,RAP1B,RRAS2 |
| ERK5 Signaling | 4,76 | 0,167 | ATF2,CREB1,FOS,MAP3K3,MEF2A,MEF2D,MYC,RAP1A,RAP1B,RRAS2,SGK1,WNK1 |
| HGF Signaling | 4,65 | 0,135 | ATF2,ATM,ETS1,FOS,ITGA2,MAP3K1,MAP3K14,MAP3K3,MAP3K9,MAPK1,PIK3R3,RAF1,RAP1A,RAP1B,RRAS2 |
| Non-Small Cell Lung Cancer Signaling | 4,64 | 0,162 | ATM,ITPR1,MAPK1,PIK3R3,RAF1,RAP1A,RAP1B,RASSF1,RASSF5,RRAS2,STK4,TP53 |
| Estrogen-Dependent Breast Cancer Signaling | 4,64 | 0,162 | ATF2,ATM,CREB1,CYP19A1,ESR1,FOS,MAPK1,PIK3R3,RAP1A,RAP1B,RRAS2,STAT5B |
| RANK Signaling in Osteoclasts | 4,58 | 0,149 | ATM,CALM1 (includes others),FOS,MAP3K1,MAP3K14,MAP3K3,MAP3K9,MAPK1,MAPK14,PIK3R3,RAF1,TNFRSF11A,XIAP |
| IL-6 Signaling | 4,58 | 0,127 | ATM,CYP19A1,FOS,MAP2K3,MAP3K14,MAPK1,MAPK14,PIK3R3,RAF1,RAP1A,RAP1B,RRAS2,SOCS1,SOCS3,TNFAIP6,  TNFRSF1B |
| Neurotrophin/TRK Signaling | 4,46 | 0,156 | ATF2,ATM,CREB1,FOS,FRS2,MAP2K3,MAPK1,PIK3R3,RAF1,RAP1A,RAP1B,RRAS2 |
| Cardiac Hypertrophy Signaling | 4,37 | 0,0975 | ADSS,ATF2,ATM,CALM1 (includes others),CREB1,MAP2K3,MAP3K1,MAP3K14,MAP3K3,MAP3K9,MAPK1,MAPK14,MEF2A,  MEF2D,PIK3R3,PRKACB,RAF1,RAP1A,RAP1B,RHOB,RRAS2,TGFB1,TGFBR2 |
| Role of NANOG in Mammalian Embryonic Stem Cell Pluripotency | 4,34 | 0,127 | ATM,BMP3,BMPR2,FZD6,MAPK1,PIK3R3,RAF1,RAP1A,RAP1B,RRAS2,SMAD4,SMAD5,TP53,WNT10A,WNT7B |
| RAR Activation | 4,32 | 0,105 | ACTB,ARID1A,FOS,GTF2H5,KAT2B,MAP3K1,MAPK1,MAPK14,PIK3R3,PNRC1,PRKACB,PTEN,RDH11,SMAD4,SMAD5,  SMARCA2,STAT5B,TAF4,TGFB1,TNIP1 |
| STAT3 Pathway | 4,21 | 0,119 | BMPR2,MAP3K9,MAPK1,MAPK14,MYC,RAF1,RAP1A,RAP1B,RRAS2,SOCS1,SOCS3,SOCS4,SOCS7,TGFB1,TGFBR2,  TNFRSF11A |
| Sertoli Cell-Sertoli Cell Junction Signaling | 4,15 | 0,105 | ACTB,ATF2,CGN,EPN2,ITGA2,MAP2K3,MAP3K1,MAP3K14,MAP3K3,MAP3K9,MAPK1,MAPK14,PRKACB,PTEN,RAB8B,RAF1,  RAP1A,RAP1B,RRAS2 |
| Aryl Hydrocarbon Receptor Signaling | 4,13 | 0,117 | ATM,CCNA2,CCND2,CHEK1,CHEK2,ESR1,ESR2,FAS,FOS,MAPK1,MYC,NCOA3,NFIA,NFIB,TGFB1,TP53 |
| PEDF Signaling | 4,13 | 0,145 | ATM,FAS,GDNF,MAPK1,MAPK14,PIK3R3,RAF1,RAP1A,RAP1B,RRAS2,TCF4,TP53 |
| Role of Osteoblasts, Osteoclasts and Chondrocytes in Rheumatoid Arthritis | 4,03 | 0,0972 | ATM,BMP3,BMPR2,CALM1 (includes others),FOS,FZD6,ITGA2,MAP2K3,MAP3K14,MAPK1,MAPK14,PIK3R3,SMAD4,SMAD5,  TCF4,TGFB1,TNFRSF11A,TNFRSF1B,WNT10A,WNT7B,XIAP |
| GNRH Signaling | 4,01 | 0,106 | ATF2,CACNB2,CALM1 (includes others),CREB1,FOS,ITPR1,MAP2K3,MAP3K1,MAP3K14,MAP3K3,MAP3K9,MAPK1,MAPK14,  PRKACB,RAF1,RAP1A,RAP1B,RRAS2 |
| Endometrial Cancer Signaling | 4 | 0,164 | ATM,MAPK1,MYC,PIK3R3,PTEN,RAF1,RAP1A,RAP1B,RRAS2,TP53 |
| IL-2 Signaling | 3,94 | 0,161 | ATM,FOS,MAPK1,PIK3R3,RAF1,RAP1A,RAP1B,RRAS2,SOCS1,STAT5B |
| PDGF Signaling | 3,92 | 0,138 | ATM,FOS,MAP3K1,MAPK1,MYC,OCRL,PIK3R3,RAF1,RAP1A,RAP1B,RASA1,RRAS2 |
| NGF Signaling | 3,92 | 0,123 | ATF2,ATM,CREB1,MAP3K1,MAP3K14,MAP3K3,MAP3K9,MAPK1,PIK3R3,RAF1,RAP1A,RAP1B,RRAS2,TP53 |
| Melanoma Signaling | 3,91 | 0,176 | ATM,MAPK1,PIK3R3,PTEN,RAF1,RAP1A,RAP1B,RRAS2,TP53 |
| Regulation of IL-2 Expression in Activated and Anergic T Lymphocytes | 3,88 | 0,136 | CALM1 (includes others),FOS,MALT1,MAP3K1,MAPK1,RAF1,RAP1A,RAP1B,RRAS2,SMAD4,TGFB1,TGFBR2 |
| Ceramide Signaling | 3,83 | 0,135 | ATM,CERK,FOS,MAP3K1,PIK3R3,PPP2R5E,RAF1,RAP1A,RAP1B,RRAS2,S1PR2,TNFRSF1B |
| Acute Myeloid Leukemia Signaling | 3,83 | 0,135 | ATM,KITLG,MAP2K3,MAPK1,MYC,PIK3R3,RAF1,RAP1A,RAP1B,RRAS2,STAT5B,TCF4 |
| Thrombopoietin Signaling | 3,82 | 0,156 | ATM,FOS,MAPK1,MYC,PIK3R3,RAF1,RAP1A,RAP1B,RRAS2,STAT5B |
| Erythropoietin Signaling | 3,79 | 0,143 | ATM,FOS,MAPK1,PIK3R3,RAF1,RAP1A,RAP1B,RRAS2,SOCS1,SOCS3,STAT5B |
| NF-κB Signaling | 3,79 | 0,102 | ATM,BMPR2,MALT1,MAP3K1,MAP3K14,MAP3K3,PIK3R3,PRKACB,RAF1,RAP1A,RAP1B,RRAS2,TGFBR2,TLR2,TNFAIP3,  TNFRSF11A,TNFRSF1B,TNIP1 |
| ErbB2-ErbB3 Signaling | 3,76 | 0,154 | ATM,MAPK1,MYC,PIK3R3,PTEN,RAF1,RAP1A,RAP1B,RRAS2,STAT5B |
| Acute Phase Response Signaling | 3,75 | 0,101 | FOS,MAP2K3,MAP3K1,MAP3K14,MAPK1,MAPK14,NR3C1,PIK3R3,RAF1,RAP1A,RAP1B,RRAS2,SOCS1,SOCS3,SOCS4,SOCS7,  TCF4,TNFRSF1B |
| Glioblastoma Multiforme Signaling | 3,74 | 0,104 | ATM,E2F8,FZD6,ITPR1,MAPK1,MYC,NF1,PIK3R3,PTEN,RAF1,RAP1A,RAP1B,RHOB,RRAS2,TP53,WNT10A,WNT7B |
| Cardiac Hypertrophy Signaling (Enhanced) | 3,71 | 0,0735 | ACVR1,ATF2,ATM,BMPR2,CALM1 (includes others),EDN1,FZD6,GDPD1,HDAC4,ITGA2,ITPR1,MAP2K3,MAP3K1,MAP3K14,  MAP3K3,MAP3K9,MAPK1,MAPK14,MEF2A,MEF2D,MYC,PDE4A,PDE4D,PIK3R3,PRKACB,PTEN,RAF1,RAP1A,RAP1B,RRAS2,  TGFB1,TGFBR2,TNFRSF1B,WNT10A,WNT7B |
| Renal Cell Carcinoma Signaling | 3,68 | 0,139 | ATM,EGLN3,ETS1,FOS,MAPK1,PIK3R3,RAF1,RAP1A,RAP1B,RRAS2,TGFB1 |
| FLT3 Signaling in Hematopoietic Progenitor Cells | 3,63 | 0,138 | ATF2,ATM,CREB1,MAPK1,MAPK14,PIK3R3,RAF1,RAP1A,RAP1B,RRAS2,STAT5B |
| Hereditary Breast Cancer Signaling | 3,6 | 0,109 | ACTB,ARID1A,ATM,CHEK1,CHEK2,FANCF,HDAC4,PIK3R3,PTEN,RAP1A,RAP1B,RRAS2,SMARCA2,TP53,WEE1 |
| EGF Signaling | 3,59 | 0,161 | ATM,FOS,ITPR1,MAP3K1,MAPK1,MAPK14,PIK3R3,RAF1,RASA1 |
| Ovarian Cancer Signaling | 3,53 | 0,108 | ATM,EDN1,FZD6,MAPK1,PIK3R3,PRKACB,PTEN,RAF1,RAP1A,RAP1B,RRAS2,TCF4,TP53,WNT10A,WNT7B |
| ATM Signaling | 3,52 | 0,125 | ATF2,ATM,CBX1,CBX5,CHEK1,CHEK2,CREB1,MAPK14,MDM4,PPP2R5E,RBBP8,TP53 |
| FAT10 Cancer Signaling Pathway | 3,49 | 0,174 | ACVR1,BMPR2,SMAD4,TCF4,TGFB1,TGFBR2,TNFRSF1B,TP53 |
| PFKFB4 Signaling Pathway | 3,49 | 0,174 | ATF2,CREB1,MAP2K3,MAPK1,NCOA3,PRKACB,TGFB1,TP53 |
| Type II Diabetes Mellitus Signaling | 3,46 | 0,106 | ACSL4,ADIPOR2,ATM,CACNB2,MAP3K1,MAP3K14,MAPK1,PIK3R3,PRKAA1,SLC27A1,SOCS1,SOCS3,SOCS4,SOCS7,TNFRSF1B |
| Apoptosis Signaling | 3,43 | 0,122 | BCL2L11,CASP10,FAS,MAP3K14,MAPK1,RAF1,RAP1A,RAP1B,RRAS2,TNFRSF1B,TP53,XIAP |
| FGF Signaling | 3,4 | 0,129 | ATF2,ATM,CREB1,FRS2,ITPR1,MAP2K3,MAP3K1,MAPK1,MAPK14,PIK3R3,RAF1 |
| Endocannabinoid Cancer Inhibition Pathway | 3,39 | 0,105 | ATF2,ATM,CASP10,CASQ1,CCND2,CREB1,MAP2K3,MAPK1,MAPK14,MYC,PIK3R3,PRKAA1,PRKACB,RAF1,TCF4 |
| Regulation of the Epithelial-Mesenchymal Transition Pathway | 3,35 | 0,0938 | ATM,ETS1,FRS2,FZD6,HMGA2,MAP2K3,MAPK1,PIK3R3,RAF1,RAP1A,RAP1B,RRAS2,SMAD4,TCF4,TGFB1,TGFBR2,WNT10A,  WNT7B |
| Apelin Endothelial Signaling Pathway | 3,33 | 0,113 | ATM,CALM1 (includes others),FOS,HDAC4,MAPK1,MEF2A,MEF2D,PIK3R3,PRKAA1,RAF1,RAP1A,RAP1B,RRAS2 |
| ERK/MAPK Signaling | 3,32 | 0,0933 | ATF2,ATM,CREB1,ESR1,ETS1,FOS,ITGA2,MAPK1,MYC,MYCN,PIK3R3,PPP2R5E,PRKACB,RAF1,RAP1A,RAP1B,RAPGEF4,RRAS2 |
| Sumoylation Pathway | 3,31 | 0,119 | ETS1,FAS,FOS,NR3C1,RAN,RCOR1,RHOB,SERBP1,SMAD4,TP53,XIAP,ZNF217 |
| Endocannabinoid Developing Neuron Pathway | 3,29 | 0,112 | ATF2,ATM,CREB1,MAP2K3,MAPK1,MAPK14,MAPK6,PIK3R3,PRKACB,RAF1,RAP1A,RAP1B,RRAS2 |
| Role of NFAT in Cardiac Hypertrophy | 3,26 | 0,0896 | ATM,CACNB2,CALM1 (includes others),HDAC4,ITPR1,MAP2K3,MAP3K1,MAPK1,MAPK14,MEF2A,MEF2D,PIK3R3,PRKACB,  RAF1,RAP1A,RAP1B,RRAS2,TGFB1,TGFBR2 |
| Cholecystokinin/Gastrin-mediated Signaling | 3,25 | 0,111 | ATF2,FOS,ITPR1,MAP2K3,MAPK1,MAPK14,MEF2A,MEF2D,RAF1,RAP1A,RAP1B,RHOB,RRAS2 |
| Thyroid Cancer Signaling | 3,23 | 0,16 | GDNF,MAPK1,MYC,RAP1A,RAP1B,RRAS2,TCF4,TP53 |
| Colorectal Cancer Metastasis Signaling | 3,23 | 0,085 | ATM,DCC,FOS,FZD6,GRK3,MAPK1,MYC,PIK3R3,PRKACB,RAP1A,RAP1B,RHOB,RRAS2,SMAD4,TCF4,TGFB1,TGFBR2,TLR2,  TP53,WNT10A,WNT7B |
| Human Embryonic Stem Cell Pluripotency | 3,22 | 0,105 | ACVR1,ATM,BMP3,BMPR2,FZD6,PIK3R3,S1PR2,SMAD4,SMAD5,TCF4,TGFB1,TGFBR2,WNT10A,WNT7B |
| Renin-Angiotensin Signaling | 3,22 | 0,11 | ATF2,ATM,FOS,ITPR1,MAP3K1,MAPK1,MAPK14,PIK3R3,PRKACB,RAF1,RAP1A,RAP1B,RRAS2 |
| EIF2 Signaling | 3,21 | 0,0888 | ACTB,AGO1,AGO3,ATM,EIF3L,EIF4A2,HNRNPA1,MAPK1,MYC,MYCN,PIK3R3,RAF1,RAP1A,RAP1B,RPL30,RPLP0,RPS4Y1,  RRAS2,XIAP |
| Role of Macrophages, Fibroblasts and Endothelial Cells in Rheumatoid Arthritis | 3,21 | 0,0797 | ATF2,ATM,CALM1 (includes others),CREB1,FOS,FZD6,MAP2K3,MAP3K14,MAPK1,MAPK14,MYC,PIK3R3,RAF1,RAP1A,RAP1B,  RRAS2,SOCS1,SOCS3,TCF4,TGFB1,TLR2,TNFRSF1B,WNT10A,WNT7B |
| Factors Promoting Cardiogenesis in Vertebrates | 3,19 | 0,122 | ACVR1,ATF2,BMP3,BMPR2,FZD6,MAPK14,SMAD4,SMAD5,TCF4,TGFB1,TGFBR2 |
| Prostate Cancer Signaling | 3,19 | 0,122 | ATF2,ATM,CREB1,MAPK1,PIK3R3,PTEN,RAF1,RAP1A,RAP1B,RRAS2,TP53 |
| UVC-Induced MAPK Signaling | 3,17 | 0,157 | FOS,MAPK1,MAPK14,RAF1,RAP1A,RAP1B,RRAS2,TP53 |
| Telomerase Signaling | 3,16 | 0,114 | ATM,ETS1,HDAC4,MAPK1,MYC,PIK3R3,PPP2R5E,RAF1,RAP1A,RAP1B,RRAS2,TP53 |
| T Cell Receptor Signaling | 3,16 | 0,114 | ATM,CALM1 (includes others),FOS,MALT1,MAP3K1,MAPK1,PIK3R3,RAF1,RAP1A,RAP1B,RASA1,RRAS2 |
| TNFR2 Signaling | 3,16 | 0,207 | FOS,MAP3K1,MAP3K14,TNFAIP3,TNFRSF1B,XIAP |
| Insulin Receptor Signaling | 3,12 | 0,103 | ATM,GRB10,MAPK1,OCRL,PIK3R3,PRKACB,PTEN,RAF1,RAP1A,RAP1B,RRAS2,SGK1,SOCS3,STXBP4 |
| CDP-diacylglycerol Biosynthesis I | 3,1 | 0,25 | ABHD5,AGPAT5,GPAM,LCLAT1,MBOAT7 |
| Estrogen Receptor Signaling | 3,09 | 0,102 | ESR1,ESR2,GTF2H5,KAT2B,MAPK1,MED12L,MED21,NCOA3,NR3C1,RAF1,RAP1A,RAP1B,RRAS2,TAF4 |
| Regulation of eIF4 and p70S6K Signaling | 3,03 | 0,0968 | AGO1,AGO3,ATM,EIF3L,EIF4A2,ITGA2,MAPK1,MAPK14,PIK3R3,PPP2R5E,RAF1,RAP1A,RAP1B,RPS4Y1,RRAS2 |
| Neuregulin Signaling | 3,03 | 0,117 | ITGA2,MAPK1,MYC,PIK3R3,PTEN,RAF1,RAP1A,RAP1B,RNF41,RRAS2,STAT5B |
| Role of BRCA1 in DNA Damage Response | 3,02 | 0,125 | ACTB,ARID1A,ATM,CHEK1,CHEK2,E2F8,FANCF,RBBP8,SMARCA2,TP53 |
| Cyclins and Cell Cycle Regulation | 3,02 | 0,125 | ATM,CCNA2,CCND2,E2F8,HDAC4,PPP2R5E,RAF1,TGFB1,TP53,WEE1 |
| Melanocyte Development and Pigmentation Signaling | 2,99 | 0,116 | ATF2,ATM,CREB1,KITLG,MAPK1,PIK3R3,PRKACB,RAF1,RAP1A,RAP1B,RRAS2 |
| FAK Signaling | 2,99 | 0,116 | ACTB,ATM,GIT2,ITGA2,MAPK1,PIK3R3,PTEN,RAF1,RAP1A,RAP1B,RRAS2 |
| IL-17 Signaling | 2,98 | 0,123 | ATF2,ATM,MAP2K3,MAP3K14,MAPK1,MAPK14,PIK3R3,RAP1A,RAP1B,RRAS2 |
| Corticotropin Releasing Hormone Signaling | 2,97 | 0,0993 | ATF2,CACNB2,CALM1 (includes others),CREB1,FOS,ITPR1,MAPK1,MAPK14,MEF2A,MEF2D,PRKACB,RAF1,RAP1A,RAP1B |
| Osteoarthritis Pathway | 2,97 | 0,087 | ATF2,BMPR2,CASP10,CASQ1,CREB1,FZD6,HDAC4,ITGA2,OCRL,PRKAA1,S1PR2,SMAD4,SMAD5,TCF4,TGFB1,TGFBR2,TLR2,  TNFRSF1B |
| Role of JAK1 and JAK3 in γc Cytokine Signaling | 2,95 | 0,132 | ATM,MAPK1,PIK3R3,RAP1A,RAP1B,RRAS2,SOCS1,SOCS3,STAT5B |
| PTEN Signaling | 2,95 | 0,103 | BCL2L11,BMPR2,ITGA2,MAPK1,OCRL,PIK3R3,PTEN,RAF1,RAP1A,RAP1B,RRAS2,TGFBR2,TNFRSF11A |
| Oncostatin M Signaling | 2,94 | 0,163 | MAPK1,PLAU,RAF1,RAP1A,RAP1B,RRAS2,STAT5B |
| Inhibition of Angiogenesis by TSP1 | 2,92 | 0,188 | MAPK1,MAPK14,TGFB1,TGFBR2,THBS1,TP53 |
| P2Y Purigenic Receptor Signaling Pathway | 2,92 | 0,102 | ATF2,ATM,CREB1,FOS,MAPK1,MYC,P2RY1,PIK3R3,PRKACB,RAF1,RAP1A,RAP1B,RRAS2 |
| Phosphatidylglycerol Biosynthesis II (Non-plastidic) | 2,9 | 0,227 | ABHD5,AGPAT5,GPAM,LCLAT1,MBOAT7 |
| NF-κB Activation by Viruses | 2,9 | 0,12 | ATM,ITGA2,MAP3K1,MAP3K14,MAPK1,PIK3R3,RAF1,RAP1A,RAP1B,RRAS2 |
| GM-CSF Signaling | 2,82 | 0,127 | ATM,ETS1,MAPK1,PIK3R3,RAF1,RAP1A,RAP1B,RRAS2,STAT5B |
| Cancer Drug Resistance By Drug Efflux | 2,8 | 0,138 | MAPK1,PIK3R3,PTEN,RAF1,RAP1A,RAP1B,RRAS2,TP53 |
| DNA Methylation and Transcriptional Repression Signaling | 2,77 | 0,176 | ARID4B,DNMT1,HIST2H4A,HIST2H4B,MBD3,MECP2 |
| IL-15 Signaling | 2,77 | 0,125 | ATM,MAPK1,MAPK14,PIK3R3,RAF1,RAP1A,RAP1B,RRAS2,STAT5B |
| Growth Hormone Signaling | 2,77 | 0,125 | ATM,FOS,MAPK1,PIK3R3,SOCS1,SOCS3,SOCS4,SOCS7,STAT5B |
| Hypoxia Signaling in the Cardiovascular System | 2,77 | 0,125 | ATF2,ATM,CREB1,EDN1,HIF1AN,PTEN,TP53,UBE2A,UBE2D3 |
| Systemic Lupus Erythematosus In T Cell Signaling Pathway | 2,74 | 0,0829 | ATF2,ATM,CASP10,CASQ1,CREB1,DNMT1,ESR1,FAS,FOS,ITPR1,MAP2K3,MAPK1,PIK3R3,PPP2R5E,RAP1A,RAP1B,RHOB,RRAS2 |
| Synaptogenesis Signaling Pathway | 2,72 | 0,0747 | AP2B1,ATF2,ATM,CALM1 (includes others),CREB1,ITPR1,LRP8,MAPK1,MAPK14,NAPB,PIK3R3,PRKACB,RAB5B,RAF1,RAP1A,  RAP1B,RRAS2,SNAP25,STX16,STXBP4,THBS1,VAMP3,WASL |
| p38 MAPK Signaling | 2,71 | 0,102 | ATF2,CREB1,FAS,MAP2K3,MAPK14,MEF2A,MEF2D,MYC,TGFB1,TGFBR2,TNFRSF1B,TP53 |
| Production of Nitric Oxide and Reactive Oxygen Species in Macrophages | 2,65 | 0,086 | ATM,FOS,MAP3K1,MAP3K14,MAP3K3,MAP3K9,MAPK1,MAPK14,PIK3R3,PPARA,PPP2R5E,RAP1A,RAP1B,RHOB,TLR2,  TNFRSF1B |
| Toll-like Receptor Signaling | 2,65 | 0,12 | FOS,MAP2K3,MAP3K1,MAP3K14,MAPK1,MAPK14,PPARA,TLR2,TNFAIP3 |
| Role of JAK family kinases in IL-6-type Cytokine Signaling | 2,64 | 0,2 | MAPK1,MAPK14,SOCS1,SOCS3,STAT5B |
| CCR3 Signaling in Eosinophils | 2,62 | 0,0992 | ATM,CALM1 (includes others),CFL2,ITPR1,MAPK1,MAPK14,MPRIP,PIK3R3,RAF1,RAP1A,RAP1B,RRAS2 |
| Cell Cycle: G2/M DNA Damage Checkpoint Regulation | 2,6 | 0,143 | ATM,CHEK1,CHEK2,KAT2B,MDM4,TP53,WEE1 |
| Chemokine Signaling | 2,57 | 0,117 | CALM1 (includes others),FOS,MAPK1,MAPK14,MPRIP,RAF1,RAP1A,RAP1B,RRAS2 |
| Antiproliferative Role of TOB in T Cell Signaling | 2,56 | 0,192 | CCNA2,MAPK1,SMAD4,TGFB1,TGFBR2 |
| Estrogen-mediated S-phase Entry | 2,56 | 0,192 | CCNA2,E2F8,ESR1,ESR2,MYC |
| Pyridoxal 5'-phosphate Salvage Pathway | 2,52 | 0,125 | GRK4,HIPK1,MAP2K3,MAP3K9,MAPK1,MAPK6,PRKAA1,SGK1 |
| HMGB1 Signaling | 2,5 | 0,0886 | ATM,FOS,KAT2A,KAT2B,MAP2K3,MAPK1,MAPK14,PIK3R3,RAP1A,RAP1B,RHOB,RRAS2,TGFB1,TNFRSF1B |
| ErbB Signaling | 2,49 | 0,106 | ATM,FOS,MAP2K3,MAPK1,MAPK14,PIK3R3,RAF1,RAP1A,RAP1B,RRAS2 |
| CD40 Signaling | 2,48 | 0,123 | ATM,FOS,MAP2K3,MAP3K14,MAPK1,MAPK14,PIK3R3,TNFAIP3 |
| Glioma Signaling | 2,47 | 0,1 | ATM,CALM1 (includes others),E2F8,MAPK1,PIK3R3,PTEN,RAF1,RAP1A,RAP1B,RRAS2,TP53 |
| AMPK Signaling | 2,46 | 0,0802 | ACTB,ARID1A,ATF2,ATM,CAB39,CCNA2,CREB1,KAT2B,MAP2K3,MAPK1,MAPK14,PIK3R3,PPP2R5E,PRKAA1,PRKACB,RAB1A,  SMARCA2 |
| IL-3 Signaling | 2,45 | 0,112 | ATM,FOS,MAPK1,PIK3R3,RAF1,RAP1A,RAP1B,RRAS2,STAT5B |
| CD27 Signaling in Lymphocytes | 2,45 | 0,135 | FOS,MAP2K3,MAP3K1,MAP3K14,MAP3K3,MAP3K9,SIVA1 |
| Myc Mediated Apoptosis Signaling | 2,44 | 0,121 | ATM,FAS,MYC,PIK3R3,RAP1A,RAP1B,RRAS2,TP53 |
| Cell Cycle: G1/S Checkpoint Regulation | 2,44 | 0,121 | ATM,CCND2,E2F8,HDAC4,MYC,SMAD4,TGFB1,TP53 |
| HIF1α Signaling | 2,41 | 0,0982 | ATM,EDN1,EGLN3,MAPK1,MAPK14,MAPK6,PIK3R3,RAP1A,RAP1B,RRAS2,TP53 |
| PAK Signaling | 2,39 | 0,103 | ATM,CFL2,ITGA2,MAPK1,PIK3R3,RAF1,RAP1A,RAP1B,RRAS2,WASL |
| UVA-Induced MAPK Signaling | 2,39 | 0,103 | ATM,FOS,MAPK1,MAPK14,PIK3R3,RAP1A,RAP1B,RRAS2,TNKS,TP53 |
| Cdc42 Signaling | 2,39 | 0,093 | ATF2,CFL2,CLIP1,EXOC7,FOS,ITGA2,MAPK1,MAPK14,MPRIP,RAF1,RASA1,WASL |
| Huntington's Disease Signaling | 2,36 | 0,0766 | ATF2,ATM,CASP10,CASQ1,CREB1,HDAC4,ITPR1,MAPK1,NAPB,PIK3R3,RASA1,RCOR1,SGK1,SNAP25,STX16,TAF4,TP53,  VAMP3 |
| IL-4 Signaling | 2,35 | 0,108 | ATM,NR3C1,NR3C2,OCRL,PIK3R3,RAP1A,RAP1B,RRAS2,SOCS1 |
| Endothelin-1 Signaling | 2,34 | 0,0824 | ATM,CASP10,CASQ1,EDN1,FOS,ITPR1,MAPK1,MAPK14,MAPK6,MYC,PIK3R3,RAF1,RAP1A,RAP1B,RRAS2 |
| Adipogenesis pathway | 2,31 | 0,0909 | ATG5,BMPR2,CLOCK,FZD6,GTF2H5,HDAC4,KAT2A,KAT2B,SMAD5,STAT5B,TGFB1,TP53 |
| Role of Tissue Factor in Cancer | 2,27 | 0,094 | ATM,CFL2,MAPK1,MAPK14,PIK3R3,PTEN,RAP1A,RAP1B,RRAS2,STAT5B,TP53 |
| PPAR Signaling | 2,27 | 0,099 | FOS,MAP3K14,MAPK1,PPARA,RAF1,RAP1A,RAP1B,RRAS2,STAT5B,TNFRSF1B |
| PKCθ Signaling in T Lymphocytes | 2,26 | 0,0861 | ATM,CACNB2,FOS,MALT1,MAP3K1,MAP3K14,MAP3K3,MAP3K9,MAPK1,PIK3R3,RAP1A,RAP1B,RRAS2 |
| PI3K Signaling in B Lymphocytes | 2,26 | 0,0896 | ATF2,CALM1 (includes others),CREB1,FOS,ITPR1,MALT1,MAPK1,PTEN,RAF1,RAP1A,RAP1B,RRAS2 |
| SAPK/JNK Signaling | 2,24 | 0,098 | ATF2,ATM,MAP3K1,MAP3K3,MAP3K9,PIK3R3,RAP1A,RAP1B,RRAS2,TP53 |
| CNTF Signaling | 2,23 | 0,123 | ATM,MAPK1,PIK3R3,RAF1,RAP1A,RAP1B,RRAS2 |
| Role of CHK Proteins in Cell Cycle Checkpoint Control | 2,23 | 0,123 | ATM,ATMIN,CHEK1,CHEK2,E2F8,PPP2R5E,TP53 |
| Wnt/β-catenin Signaling | 2,22 | 0,0824 | ACVR1,BMPR2,CSNK1G1,FZD6,MYC,PPP2R5E,SOX4,SOX6,TCF4,TGFB1,TGFBR2,TP53,WNT10A,WNT7B |
| Opioid Signaling Pathway | 2,2 | 0,0738 | AP2B1,ATF2,CACNB2,CALM1 (includes others),CREB1,FOS,GRK3,GRK4,ITPR1,MAP2K3,MAPK1,MAPK6,MYC,PRKACB,RAF1,  RAP1A,RAP1B,RRAS2 |
| Glioma Invasiveness Signaling | 2,17 | 0,11 | ATM,MAPK1,PIK3R3,PLAU,RAP1A,RAP1B,RHOB,RRAS2 |
| Role of JAK2 in Hormone-like Cytokine Signaling | 2,15 | 0,156 | SOCS1,SOCS3,SOCS4,SOCS7,STAT5B |
| Purine Nucleotides De Novo Biosynthesis II | 2,13 | 0,273 | ADSS,IMPDH1,PAICS |
| Angiopoietin Signaling | 2,1 | 0,107 | ATM,PIK3R3,RAP1A,RAP1B,RASA1,RRAS2,STAT5B,TNIP1 |
| CDK5 Signaling | 2,09 | 0,0935 | ITGA2,MAPK1,MAPK14,MAPK6,PPP2R5E,PRKACB,RAF1,RAP1A,RAP1B,RRAS2 |
| Type I Diabetes Mellitus Signaling | 2,09 | 0,0935 | FAS,MAP2K3,MAP3K14,MAPK1,MAPK14,SOCS1,SOCS3,SOCS4,SOCS7,TNFRSF1B |
| Paxillin Signaling | 2,09 | 0,0935 | ACTB,ATM,GIT2,ITGA2,MAPK1,MAPK14,PIK3R3,RAP1A,RAP1B,RRAS2 |
| Role of NFAT in Regulation of the Immune Response | 2,07 | 0,0791 | ATF2,ATM,CALM1 (includes others),CSNK1G1,FOS,ITPR1,MAPK1,MEF2A,MEF2D,PIK3R3,RAF1,RAP1A,RAP1B,RRAS2 |
| Role of MAPK Signaling in the Pathogenesis of Influenza | 2,07 | 0,105 | ATF2,MAP2K3,MAPK1,MAPK14,RAF1,RAP1A,RAP1B,RRAS2 |
| Polyamine Regulation in Colon Cancer | 2,04 | 0,182 | AZIN1,MXD1,MYC,TCF4 |
| IL-9 Signaling | 2,04 | 0,147 | ATM,BCL3,PIK3R3,SOCS3,STAT5B |
| SPINK1 General Cancer Pathway | 2,03 | 0,113 | ATM,MAPK1,PIK3R3,RAF1,RAP1A,RAP1B,RRAS2 |
| Rac Signaling | 1,98 | 0,0901 | ATM,CFL2,ITGA2,MAP3K1,MAPK1,PIK3R3,RAF1,RAP1A,RAP1B,RRAS2 |
| Bladder Cancer Signaling | 1,94 | 0,0938 | MAPK1,MYC,RAF1,RAP1A,RAP1B,RASSF1,RRAS2,THBS1,TP53 |
| PI3K/AKT Signaling | 1,94 | 0,0846 | ITGA2,MAPK1,OCRL,PIK3R3,PPP2R5E,PTEN,RAF1,RAP1A,RAP1B,RRAS2,TP53 |
| Gα12/13 Signaling | 1,91 | 0,084 | ATM,MAP3K1,MAPK1,MEF2A,MEF2D,PIK3R3,RAF1,RAP1A,RAP1B,RASA1,RRAS2 |
| Iron homeostasis signaling pathway | 1,91 | 0,084 | ATP6V0E1,ATP6V1B2,ATP6V1C1,BMP3,BMPR2,MAPK1,SLC46A1,SLC48A1,SMAD4,SMAD5,STAT5B |
| Tight Junction Signaling | 1,91 | 0,0778 | ACTB,CGN,FOS,NAPB,PPP2R5E,PRKACB,PTEN,SNAP25,STX16,TGFB1,TGFBR2,TNFRSF1B,VAMP3 |
| IL-22 Signaling | 1,91 | 0,167 | MAPK1,MAPK14,SOCS3,STAT5B |
| Epithelial Adherens Junction Signaling | 1,89 | 0,08 | ACTB,ACVR1,BMPR2,CLIP1,EPN2,PTEN,RAP1A,RAP1B,RRAS2,TCF4,TGFBR2,WASL |
| Protein Kinase A Signaling | 1,88 | 0,0627 | AKAP2,ATF2,CALM1 (includes others),CREB1,DCC,GDPD1,ITPR1,MAP3K1,MAPK1,PDE4A,PDE4D,PRKACB,PTEN,PTP4A1,  PTPN4,PTPRB,PTPRG,RAF1,RAP1A,RAP1B,SMAD4,TCF4,TGFB1,TGFBR2 |
| Sirtuin Signaling Pathway | 1,87 | 0,0671 | ATG14,ATG16L1,ATG2B,ATG5,BCL2L11,CLOCK,KAT2A,MAPK1,MAPK6,MYC,MYCN,NDRG1,NDUFB2,PGK1,PPARA,PRKAA1,  RBBP8,SF3A1,TP53 |
| Integrin Signaling | 1,87 | 0,0728 | ACTB,ATM,ITGA2,MAPK1,MPRIP,PFN1,PFN2,PIK3R3,PTEN,RAF1,RAP1A,RAP1B,RHOB,RRAS2,WASL |
| Apelin Cardiomyocyte Signaling Pathway | 1,86 | 0,0909 | ATM,ITPR1,MAPK1,MAPK14,MAPK6,PIK3R3,SLC9A1,SLC9A6,TGFB1 |
| ErbB4 Signaling | 1,85 | 0,104 | ATM,MAPK1,PIK3R3,RAF1,RAP1A,RAP1B,RRAS2 |
| T Cell Exhaustion Signaling Pathway | 1,85 | 0,0765 | ACVR1,ATM,BMPR2,FOS,FOXP1,MAPK1,PIK3R3,PPP2R5E,RAP1A,RAP1B,RRAS2,TGFB1,TGFBR2 |
| Triacylglycerol Biosynthesis | 1,84 | 0,132 | ABHD5,AGPAT5,GPAM,LCLAT1,MBOAT7 |
| Fc Epsilon RI Signaling | 1,81 | 0,0847 | ATM,MAP2K3,MAPK1,MAPK14,OCRL,PIK3R3,RAF1,RAP1A,RAP1B,RRAS2 |
| UVB-Induced MAPK Signaling | 1,81 | 0,113 | ATM,FOS,MAPK1,MAPK14,PIK3R3,TP53 |
| April Mediated Signaling | 1,79 | 0,128 | FOS,MAP3K1,MAP3K14,MAPK1,MAPK14 |
| VEGF Family Ligand-Receptor Interactions | 1,79 | 0,0941 | ATM,FOS,MAPK1,PIK3R3,RAF1,RAP1A,RAP1B,RRAS2 |
| Adrenomedullin signaling pathway | 1,77 | 0,0725 | ATM,CALM1 (includes others),FOS,ITPR1,MAP2K3,MAPK1,MAPK14,MAPK6,PIK3R3,PRKACB,RAF1,RAP1A,RAP1B,RRAS2 |
| RhoA Signaling | 1,74 | 0,0826 | ACTB,ARHGAP1,ARHGAP12,CFL2,CIT,MPRIP,PFN1,PFN2,RAPGEF2,RAPGEF6 |
| Actin Cytoskeleton Signaling | 1,74 | 0,0701 | ACTB,ATM,CFL2,ITGA2,MAPK1,MPRIP,PFN1,PFN2,PIK3R3,RAF1,RAP1A,RAP1B,RRAS2,SLC9A1,WASL |
| Role of IL-17A in Arthritis | 1,74 | 0,109 | ATF2,ATM,MAP2K3,MAPK1,MAPK14,PIK3R3 |
| Superpathway of Cholesterol Biosynthesis | 1,73 | 0,148 | HADHB,HMGCS1,LBR,MSMO1 |
| Phagosome Maturation | 1,72 | 0,0786 | ATP6V0E1,ATP6V1B2,ATP6V1C1,DYNC1LI2,NAPB,RAB5B,SNAP25,STX16,VAMP3,VPS37A,VPS37B |
| B Cell Activating Factor Signaling | 1,71 | 0,122 | FOS,MAP3K1,MAP3K14,MAPK1,MAPK14 |
| Zymosterol Biosynthesis | 1,7 | 0,333 | LBR,MSMO1 |
| Calcium Signaling | 1,68 | 0,0707 | ATF2,CACNB2,CALM1 (includes others),CASQ1,CREB1,HDAC4,ITPR1,MAPK1,MEF2A,MEF2D,PRKACB,RAP1A,RAP1B,TRPC3 |
| BAG2 Signaling Pathway | 1,67 | 0,119 | MAPK1,MAPK14,MYC,PRKN,TP53 |
| Superpathway of Geranylgeranyldiphosphate Biosynthesis I (via Mevalonate) | 1,66 | 0,188 | COX10,HADHB,HMGCS1 |
| Parkinson's Signaling | 1,66 | 0,188 | MAPK1,MAPK14,PRKN |
| Death Receptor Signaling | 1,65 | 0,0889 | ACTB,CASP10,FAS,MAP3K14,TNFRSF10B,TNFRSF1B,TNKS,XIAP |
| Virus Entry via Endocytic Pathways | 1,64 | 0,0833 | ACTB,AP2B1,AP3B1,ATM,ITGA2,PIK3R3,RAP1A,RAP1B,RRAS2 |
| IL-7 Signaling Pathway | 1,63 | 0,0946 | ATM,MAPK1,MAPK14,MYC,PIK3R3,SOCS1,STAT5B |
| Synaptic Long Term Potentiation | 1,63 | 0,0794 | ATF2,CALM1 (includes others),CREB1,ITPR1,MAPK1,PRKACB,RAF1,RAP1A,RAP1B,RRAS2 |
| Role of p14/p19ARF in Tumor Suppression | 1,62 | 0,138 | ATM,PIK3R3,SF3A1,TP53 |
| Pancreatic Adenocarcinoma Signaling | 1,61 | 0,0826 | ATM,E2F8,MAPK1,PIK3R3,RAF1,SMAD4,TGFB1,TGFBR2,TP53 |
| Relaxin Signaling | 1,58 | 0,0748 | ATM,CREB1,FOS,GDPD1,MAPK1,PDE4A,PDE4D,PIK3R3,PRKACB,RAP1A,RAP1B |
| α-Adrenergic Signaling | 1,58 | 0,086 | CALM1 (includes others),ITPR1,MAPK1,PRKACB,RAF1,RAP1A,RAP1B,RRAS2 |
| PCP pathway | 1,57 | 0,1 | ATF2,FZD6,PFN1,PFN2,WNT10A,WNT7B |
| ILK Signaling | 1,56 | 0,0699 | ACTB,ATF2,ATM,CFL2,CREB1,FBLIM1,FOS,MAPK1,MYC,PIK3R3,PPP2R5E,PTEN,RHOB |
| NRF2-mediated Oxidative Stress Response | 1,54 | 0,0695 | ACTB,ATM,DNAJA2,FOS,MAP2K3,MAP3K1,MAPK1,MAPK14,PIK3R3,RAF1,RAP1A,RAP1B,RRAS2 |
| Salvage Pathways of Pyrimidine Ribonucleotides | 1,53 | 0,0842 | GRK4,HIPK1,MAP2K3,MAP3K9,MAPK1,MAPK6,PRKAA1,SGK1 |
| Reelin Signaling in Neurons | 1,5 | 0,0886 | ATM,ITGA2,LRP8,MAP3K9,NDEL1,PAFAH1B2,PIK3R3 |
| 4-1BB Signaling in T Lymphocytes | 1,48 | 0,125 | ATF2,MAP3K14,MAPK1,MAPK14 |
| Th2 Pathway | 1,47 | 0,0746 | ACVR1,ATM,BMPR2,CXCR6,IKZF1,PIK3R3,SOCS3,STAT5B,TGFB1,TGFBR2 |
| Cardiomyocyte Differentiation via BMP Receptors | 1,46 | 0,158 | ATF2,BMPR2,SMAD4 |
| GADD45 Signaling | 1,46 | 0,158 | ATM,CCND2,TP53 |
| fMLP Signaling in Neutrophils | 1,46 | 0,0776 | ATM,CALM1 (includes others),ITPR1,MAPK1,PIK3R3,RAF1,RAP1A,RAP1B,RRAS2 |
| VEGF Signaling | 1,46 | 0,0816 | ACTB,ATM,MAPK1,PIK3R3,RAF1,RAP1A,RAP1B,RRAS2 |
| Regulation of Cellular Mechanics by Calpain Protease | 1,45 | 0,0938 | CCNA2,ITGA2,MAPK1,RAP1A,RAP1B,RRAS2 |
| TNFR1 Signaling | 1,44 | 0,104 | FOS,MAP3K1,MAP3K14,TNFAIP3,XIAP |
| Leukocyte Extravasation Signaling | 1,43 | 0,067 | ACTB,ARHGAP1,ARHGAP12,ATM,ITGA2,MAPK1,MAPK14,PIK3R3,RAP1A,RAP1B,RAPGEF4,RASSF5,WASL |
| Gap Junction Signaling | 1,42 | 0,0667 | ACTB,ATM,CSNK1G1,DBN1,ITPR1,MAPK1,PIK3R3,PRKACB,RAF1,RAP1A,RAP1B,RRAS2,SGSM3 |
| Ephrin Receptor Signaling | 1,38 | 0,0674 | ATF2,CFL2,CREB1,ITGA2,MAP3K14,MAPK1,RAF1,RAP1A,RAP1B,RASA1,RRAS2,WASL |
| HIPPO signaling | 1,37 | 0,0833 | DLG5,FRMD6,PPP2R5E,RASSF1,SMAD4,SMAD5,STK4 |
| Breast Cancer Regulation by Stathmin1 | 1,34 | 0,065 | ATM,CALM1 (includes others),E2F8,ITPR1,MAPK1,PIK3R3,PPP2R5E,PRKACB,RAF1,RAP1A,RAP1B,RRAS2,TP53 |
| IL-10 Signaling | 1,31 | 0,087 | FOS,MAP2K3,MAP3K14,MAPK1,MAPK14,SOCS3 |
