# Supplementary material for: The Long-Term Effects of Early Life Stress on the Modulation of miR-19 Levels
Source: Front Psychiatry. 2020 May 15;11:389. doi: 10.3389/fpsyt.2020.00389 (PMC7243913; doi:10.3389/fpsyt.2020.00389)
Supplement: Supplementary Table 7 — List of 198 common significant pathways regulated by miR-19a, miR-19b-1 and miR-19b-2. [file Table_7.docx]

**Supplementary Table 7.**

| 198 common significant pathways |
| --- |
| Molecular Mechanisms of Cancer |
| Germ Cell-Sertoli Cell Junction Signaling |
| B Cell Receptor Signaling |
| TGF-β Signaling |
| p53 Signaling |
| Chronic Myeloid Leukemia Signaling |
| Mouse Embryonic Stem Cell Pluripotency |
| PPARα/RXRα Activation |
| HGF Signaling |
| IL-6 Signaling |
| Endometrial Cancer Signaling |
| NF-κB Signaling |
| Non-Small Cell Lung Cancer Signaling |
| RANK Signaling in Osteoclasts |
| Melanoma Signaling |
| Glioblastoma Multiforme Signaling |
| Ceramide Signaling |
| Acute Myeloid Leukemia Signaling |
| Sertoli Cell-Sertoli Cell Junction Signaling |
| Role of NANOG in Mammalian Embryonic Stem Cell Pluripotency |
| IGF-1 Signaling |
| ErbB2-ErbB3 Signaling |
| Glucocorticoid Receptor Signaling |
| JAK/Stat Signaling |
| Prolactin Signaling |
| RAR Activation |
| PTEN Signaling |
| BMP signaling pathway |
| Apoptosis Signaling |
| Acute Phase Response Signaling |
| ERK5 Signaling |
| Role of Osteoblasts, Osteoclasts and Chondrocytes in Rheumatoid Arthritis |
| IL-2 Signaling |
| TNFR2 Signaling |
| Aryl Hydrocarbon Receptor Signaling |
| Hereditary Breast Cancer Signaling |
| Erythropoietin Signaling |
| GDNF Family Ligand-Receptor Interactions |
| Ovarian Cancer Signaling |
| Colorectal Cancer Metastasis Signaling |
| Type II Diabetes Mellitus Signaling |
| Neuregulin Signaling |
| Cardiac Hypertrophy Signaling (Enhanced) |
| PEDF Signaling |
| EGF Signaling |
| NGF Signaling |
| GM-CSF Signaling |
| Sumoylation Pathway |
| Cancer Drug Resistance By Drug Efflux |
| Production of Nitric Oxide and Reactive Oxygen Species in Macrophages |
| IL-15 Signaling |
| PDGF Signaling |
| STAT3 Pathway |
| GNRH Signaling |
| Regulation of IL-2 Expression in Activated and Anergic T Lymphocytes |
| Insulin Receptor Signaling |
| Estrogen-Dependent Breast Cancer Signaling |
| Telomerase Signaling |
| Toll-like Receptor Signaling |
| Estrogen-mediated S-phase Entry |
| Neurotrophin/TRK Signaling |
| Thyroid Cancer Signaling |
| Thrombopoietin Signaling |
| Cardiac Hypertrophy Signaling |
| Glioma Signaling |
| ErbB Signaling |
| UVC-Induced MAPK Signaling |
| FAK Signaling |
| FLT3 Signaling in Hematopoietic Progenitor Cells |
| Role of Macrophages, Fibroblasts and Endothelial Cells in Rheumatoid Arthritis |
| PI3K/AKT Signaling |
| Apelin Endothelial Signaling Pathway |
| LPS-stimulated MAPK Signaling |
| NF-κB Activation by Viruses |
| Endocannabinoid Developing Neuron Pathway |
| Cholecystokinin/Gastrin-mediated Signaling |
| Role of Tissue Factor in Cancer |
| FGF Signaling |
| PPAR Signaling |
| Renin-Angiotensin Signaling |
| Oncostatin M Signaling |
| Regulation of the Epithelial-Mesenchymal Transition Pathway |
| Regulation of eIF4 and p70S6K Signaling |
| Estrogen Receptor Signaling |
| AMPK Signaling |
| Role of NFAT in Cardiac Hypertrophy |
| CNTF Signaling |
| ERK/MAPK Signaling |
| EIF2 Signaling |
| Inhibition of Angiogenesis by TSP1 |
| T Cell Receptor Signaling |
| Prostate Cancer Signaling |
| FAT10 Cancer Signaling Pathway |
| Role of MAPK Signaling in the Pathogenesis of Influenza |
| Sirtuin Signaling Pathway |
| Chemokine Signaling |
| Melanocyte Development and Pigmentation Signaling |
| Tight Junction Signaling |
| IL-22 Signaling |
| Renal Cell Carcinoma Signaling |
| Pyridoxal 5'-phosphate Salvage Pathway |
| Bladder Cancer Signaling |
| Role of BRCA1 in DNA Damage Response |
| IL-3 Signaling |
| Adipogenesis pathway |
| PAK Signaling |
| UVA-Induced MAPK Signaling |
| IL-17 Signaling |
| Role of JAK family kinases in IL-6-type Cytokine Signaling |
| Myc Mediated Apoptosis Signaling |
| Adrenomedullin signaling pathway |
| CD27 Signaling in Lymphocytes |
| ErbB4 Signaling |
| Fc Epsilon RI Signaling |
| Role of JAK1 and JAK3 in γc Cytokine Signaling |
| UVB-Induced MAPK Signaling |
| HMGB1 Signaling |
| CCR3 Signaling in Eosinophils |
| Opioid Signaling Pathway |
| Endocannabinoid Cancer Inhibition Pathway |
| Growth Hormone Signaling |
| CDK5 Signaling |
| Type I Diabetes Mellitus Signaling |
| Death Receptor Signaling |
| Integrin Signaling |
| P2Y Purigenic Receptor Signaling Pathway |
| ILK Signaling |
| IL-7 Signaling Pathway |
| Angiopoietin Signaling |
| Cdc42 Signaling |
| HIF1α Signaling |
| Iron homeostasis signaling pathway |
| PKCθ Signaling in T Lymphocytes |
| Human Embryonic Stem Cell Pluripotency |
| ATM Signaling |
| CDP-diacylglycerol Biosynthesis I |
| SPINK1 General Cancer Pathway |
| PI3K Signaling in B Lymphocytes |
| Systemic Lupus Erythematosus In T Cell Signaling Pathway |
| Cyclins and Cell Cycle Regulation |
| TNFR1 Signaling |
| Regulation of Cellular Mechanics by Calpain Protease |
| Synaptogenesis Signaling Pathway |
| p38 MAPK Signaling |
| IL-9 Signaling |
| DNA Methylation and Transcriptional Repression Signaling |
| CD40 Signaling |
| Purine Nucleotides De Novo Biosynthesis II |
| Cell Cycle: G1/S Checkpoint Regulation |
| Phosphatidylglycerol Biosynthesis II (Non-plastidic) |
| RhoA Signaling |
| Corticotropin Releasing Hormone Signaling |
| Endothelin-1 Signaling |
| VEGF Family Ligand-Receptor Interactions |
| Osteoarthritis Pathway |
| IL-10 Signaling |
| Paxillin Signaling |
| Huntington's Disease Signaling |
| April Mediated Signaling |
| Pancreatic Adenocarcinoma Signaling |
| Wnt/β-catenin Signaling |
| Actin Cytoskeleton Signaling |
| Epithelial Adherens Junction Signaling |
| Glioma Invasiveness Signaling |
| Rac Signaling |
| Gα12/13 Signaling |
| Antiproliferative Role of TOB in T Cell Signaling |
| Role of CHK Proteins in Cell Cycle Checkpoint Control |
| α-Adrenergic Signaling |
| B Cell Activating Factor Signaling |
| BAG2 Signaling Pathway |
| Salvage Pathways of Pyrimidine Ribonucleotides |
| VEGF Signaling |
| Apelin Cardiomyocyte Signaling Pathway |
| Phagosome Maturation |
| Parkinson's Signaling |
| PFKFB4 Signaling Pathway |
| NRF2-mediated Oxidative Stress Response |
| SAPK/JNK Signaling |
| IL-4 Signaling |
| Role of JAK2 in Hormone-like Cytokine Signaling |
| T Cell Exhaustion Signaling Pathway |
| Cell Cycle: G2/M DNA Damage Checkpoint Regulation |
| Leukocyte Extravasation Signaling |
| GADD45 Signaling |
| Factors Promoting Cardiogenesis in Vertebrates |
| Role of NFAT in Regulation of the Immune Response |
| Breast Cancer Regulation by Stathmin1 |
| Ephrin Receptor Signaling |
| Hypoxia Signaling in the Cardiovascular System |
| fMLP Signaling in Neutrophils |
| Triacylglycerol Biosynthesis |
| Polyamine Regulation in Colon Cancer |
| PCP pathway |
| Relaxin Signaling |
| Gap Junction Signaling |
| Synaptic Long Term Potentiation |
| HIPPO signaling |
